# Sustainable zeolite synthesis: advances in green strategies and AI-assisted design

**DOI:** 10.3389/fchem.2026.1910454

**Published:** 2026-09-10

**Authors:** Beidi Li, Nur Hidayatul Nazirah Kamarudin, Wan Nor Roslam Wan Isahak, Noorashikin Md Saleh, Yaqin Tian

**Affiliations:** 1 Department of Chemical and Process Engineering, Faculty of Engineering and Built Environment, Universiti Kebangsaan Malaysia, Bangi, Selangor, Malaysia; 2 College of Energy and Building Engineering, Shandong Huayu University of Technology, Dezhou, Shandong, China

**Keywords:** artificial intelligence design, green synthesis chemistry, ionothermal synthesis, renewable silicon and aluminum, zeolite

## Abstract

Zeolites are porous materials with regular nanochannels, high surface area, tunable acid sites, excellent thermal and hydrothermal stability, and outstanding catalytic performance. These properties make them indispensable in energy and chemical engineering, environmental remediation, selective adsorption, and clean energy technologies. However, conventional hydrothermal synthesis suffers from high energy consumption, strong alkali corrosion, excessive template usage, and the generation of salt containing and organic waste liquids, limiting its sustainability. Consequently, green chemistry based synthesis strategies have become a major research focus, aiming to optimize resource utilization, environmental impact, and material performance simultaneously. This review summarizes recent advances in green zeolite synthesis, including solvent free or low solvent methods, low template or template free strategies, directed agent synthesis, ionothermal synthesis, dry gel conversion, and the use of renewable silicon and aluminum resources. The underlying reaction mechanisms and performance control principles are critically discussed. Through representative case studies, the review highlights how these approaches reduce activation energy, improve atom efficiency, minimize waste generation, and enable precise control of crystal morphology and pore structures. Future perspectives include AI-driven materials design, accurate topology prediction, machine learning-assisted structural characterization and generation, and the optimization of synthesis conditions.

## 1 Introduction

Since the early 1990s, scientists have gradually come to realize that certain chemicals and chemical manufacturing processes can harm human health and the natural environment. To address this issue at its source, the concept of “green chemistry” was proposed and has subsequently gained widespread acceptance (Anastas and Kirchhoff, 2002; Trost, 1991). As this concept has evolved, the search for environmentally friendly and sustainable methods to synthesize zeolites has become one of the primary focuses of zeolite related research. Zeolites are important microporous crystalline materials, and their synthesis has a history spanning more than 60 years. Traditional hydrothermal synthesis techniques have matured, but they impose a significant environmental burden. These issues include high energy consumption, highly alkaline reaction conditions, the need for large quantities of organic structure-directing agents (e.g., tetrapropylammonium hydroxide, TPAOH; tetraethylammonium hydroxide, TEAOH), and the generation of wastewater with high salt content and high chemical oxygen demand (COD). Against this backdrop, the “green synthesis” of zeolites has emerged. Its primary objective is to reduce the environmental impact of the synthesis process by minimizing energy consumption, waste generation, and the release of hazardous chemicals. By focusing on atom economy and utilizing renewable raw materials to optimize resource utilization while maintaining or even enhancing material performance, this approach propels zeolite chemistry toward a greener and more sustainable future. Accordingly, the development of greener and more resource-efficient zeolite synthesis routes has become an important research priority.

Zeolite synthesis can be considered from two main perspectives: raw materials and synthesis methods. The major raw materials include silica sources (such as silica, silica sol, and silicate esters), aluminum sources (such as sodium aluminate, aluminum sulfate, and aluminum isopropoxide), alkali sources, metal cations, mineralizing agents (such as OH^−^ and F^−^), templates (such as nitrogen-containing organics and quaternary ammonium salts), and solvents (such as water and ethanol). The primary synthesis methods include hydrothermal synthesis, ionothermal synthesis, and dry gel conversion synthesis. To address the environmental challenges associated with conventional synthesis routes, increasing attention has been devoted to green synthesis strategies, including the utilization of renewable feedstocks, the reduction or elimination of organic templates, and the optimization of synthesis processes.

Several representative review articles published in the past 5 years have summarized individual aspects of sustainable zeolite synthesis, including renewable raw materials, framework engineering, and specific green synthesis strategies. A comparison of these previous reviews with the present work is provided in Table 1.

**TABLE 1:** Comparison of representative review articles on green zeolite synthesis published over the past 5 years with the present review.

Ref.	Main focus	Core findings	Limitations
He et al. (2021)	Green synthesis of zeolites from kaolin	Reviewed sustainable synthesis routes using kaolin as a renewable aluminosilicate source, including hydrothermal, microwave, and *in situ* crystallization methods, together with synthesis mechanisms and future trends	Limited to kaolin-derived zeolites; AI-assisted design, techno-economic analysis, and industrial scale-up were not discussed
Lin et al. (2022)	Fly ash-derived zeolite synthesis	Summarized synthesis methods, reaction mechanisms, wastewater treatment applications, and resource utilization of fly ash-derived zeolites, highlighting waste valorization	Focused mainly on fly ash utilization and environmental applications, without covering intelligent synthesis or broader green synthesis strategies
Zhu et al. (2026)	Engineering zeolite synthesis for green energy transformation	Discussed recent advances in greener zeolite synthesis, multifunctional catalyst design, computational prediction, machine learning, and continuous-flow synthesis for energy applications	Focused on energy-related catalytic applications, with relatively limited discussion of renewable raw materials, ionothermal synthesis, electrochemical synthesis, and comprehensive green chemistry strategies
Sun et al. (2026)	Dual-route accessible zeolites	Proposed the concept of dual-route accessible zeolites to bridge OSDA-assisted and OSDA-free synthesis, providing mechanistic insights into crystallization and guiding OSDA-free synthesis design	Mainly focused on OSDA-free synthesis mechanisms and crystallization principles, without covering other green synthesis technologies or AI-driven synthesis optimization
This review	Comprehensive green zeolite synthesis and AI-assisted design	Systematically summarizes solvent-free/low-solvent hydrothermal synthesis, template-free synthesis, ionothermal synthesis, dry gel conversion, electrochemical synthesis, renewable silicon/aluminum resources, and machine learning-guided topology prediction, synthesis optimization, and structural characterization. Techno-economic considerations and industrial scale-up challenges are also discussed to provide an integrated perspective for sustainable zeolite manufacturing	Provides a comprehensive overview linking green chemistry, intelligent materials design, and industrial implementation. Quantitative techno-economic analysis, life-cycle assessment, and pilot-scale industrial data remain limited

Despite the significant progress achieved in sustainable zeolite synthesis, a comprehensive review that systematically integrates green synthesis strategies with emerging artificial intelligence (AI)-assisted materials design remains lacking. In this review, we provide a critical overview of recent advances in green zeolite synthesis, including solvent-free and low-solvent hydrothermal synthesis, template-free strategies, ionothermal synthesis, dry gel conversion, electrochemical synthesis, and the utilization of renewable silicon and aluminum resources. Particular emphasis is placed on the reaction mechanisms, sustainability benefits, and synthesis-structure relationships of these approaches.

**FIGURE 1:**
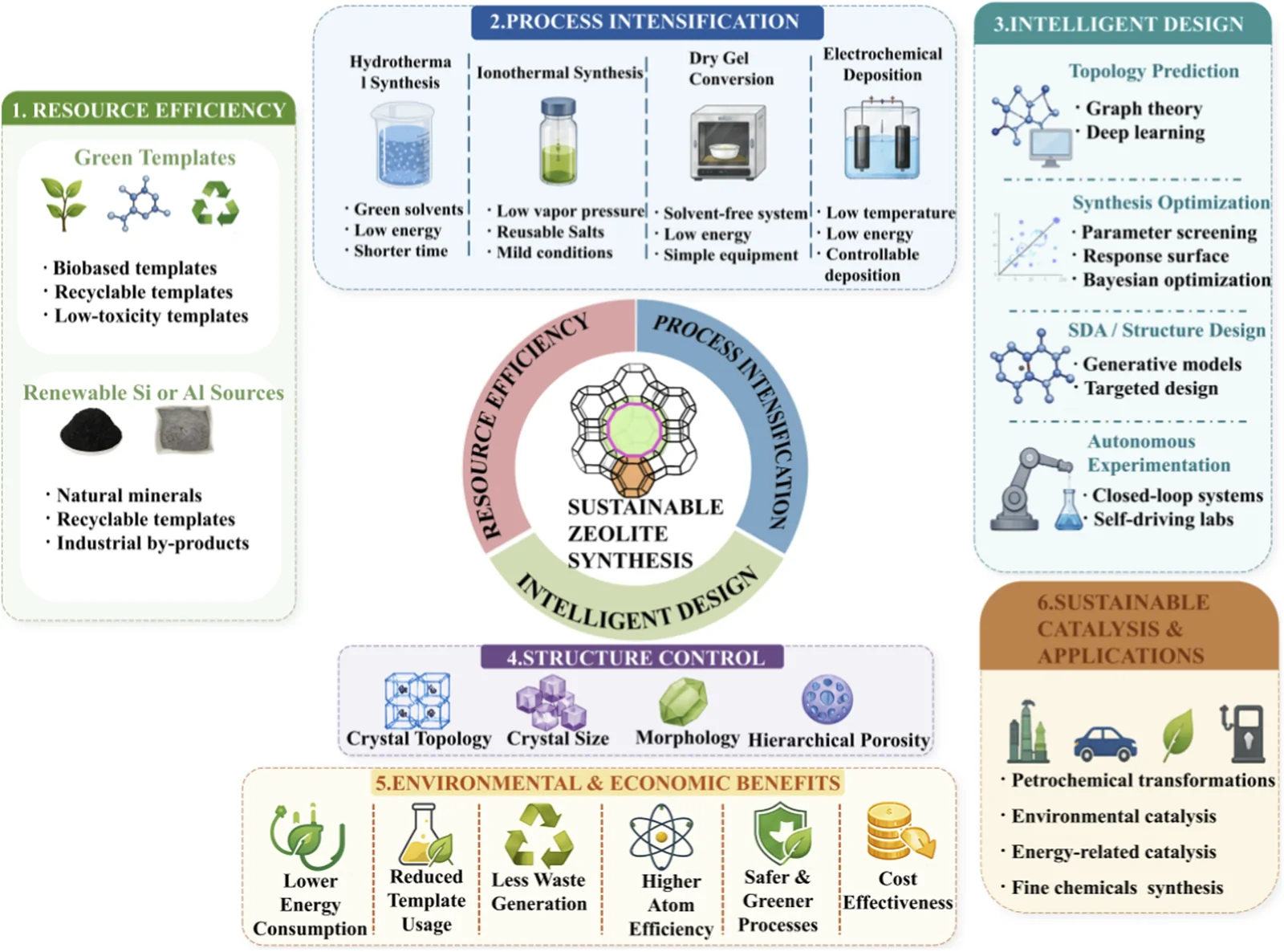
Conceptual framework of sustainable zeolite synthesis.

Furthermore, we discuss the rapidly growing role of AI and machine learning in topology prediction, synthesis optimization, structural characterization, and closed-loop materials discovery. By integrating sustainable synthesis methodologies with data-driven design strategies, this review provides a comprehensive framework and future perspectives for the sustainable development of sustainable zeolite synthesis. These green synthesis strategies provide the experimental basis for the AI-assisted materials design strategies discussed in Section 7, thereby establishing a close connection between sustainable synthesis and data-driven materials discovery. The overall framework and organization of this review are illustrated in Figure 1.

## 2 Hydrothermal synthesis

Among the established routes for zeolite synthesis, hydrothermal crystallization remains the most conventional and widely applied method. Among them, hydrothermal crystallization, as the most conventional and widely applied route, remains dominant. However, this method has several notable drawbacks. This reaction requires a large amount of water as a solvent, resulting in high water consumption and low synthesis efficiency. Moreover, the use of organic structure-directing agents (OSDAs) not only increases the cost of raw materials, but also requires a high temperature calcination for template removal, resulting in additional energy consumption and environmental impact. In addition, high temperature and high pressure synthesis conditions present considerable safety risks. As a result, more and more research is being devoted to the development of green and sustainable pathways for zeolite synthesis. These efforts are aimed at reducing solvent consumption and reaction pressure, improving synthesis efficiency, minimizing waste generation, and ultimately making the zeolite synthesis process more environmentally friendly.

**FIGURE 2:**
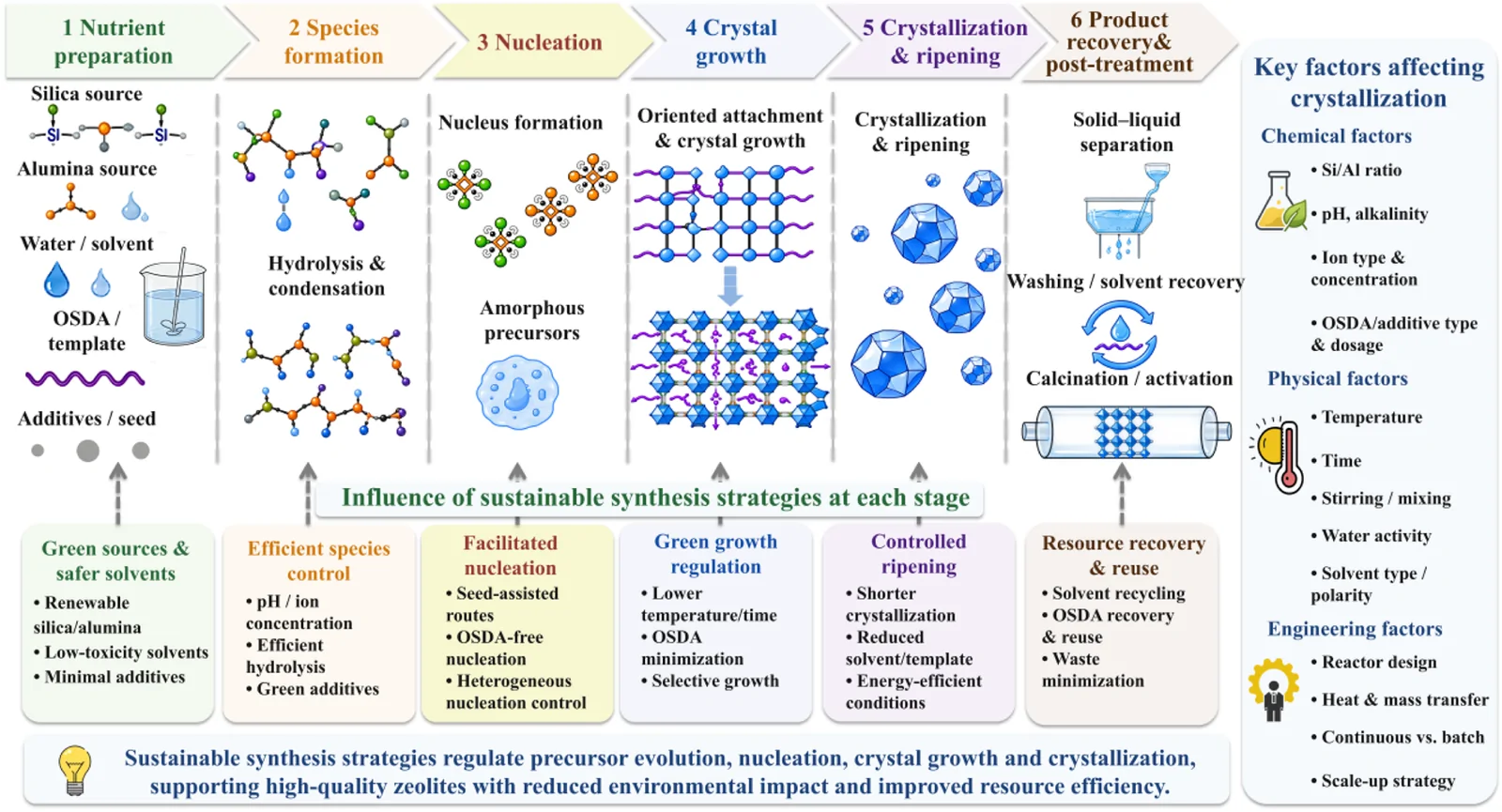
General crystallization mechanism of sustainable zeolite synthesis.

To rationally develop these sustainable synthesis strategies, it is essential to understand how changes in the synthesis environment affect zeolite crystallization. In general, zeolite crystallization involves precursor dissolution and speciation, hydrolysis and condensation of silicate and aluminate species, nucleation, crystal growth and framework assembly, followed by crystallization and ripening (Figure 2). These interconnected processes are strongly influenced by chemical parameters, including the Si/Al ratio, alkalinity, ionic composition, and structure-directing agents or additives, as well as physical parameters such as temperature, crystallization time, mixing, water activity, and solvent properties. Green synthesis strategies therefore not only reduce the consumption of solvents, templates, and conventional raw materials but may also modify precursor evolution, nucleation kinetics, mass transport, and crystal-growth pathways. Importantly, these changes can affect the crystallinity, phase selectivity, framework formation, crystal size and morphology, and defect formation of the resulting zeolites. Sustainable synthesis should therefore balance reductions in energy, solvent, and template consumption with effective control over crystallization and structural quality.

Based on these general crystallization principles, various sustainable hydrothermal strategies have been developed to regulate the synthesis environment while reducing resource consumption and environmental impacts, as discussed in the following subsections.

### 2.1 Synthesis with green templates

Organic templates are mainly used in the synthesis of zeolites (Chen et al., 2024; Ma et al., 2024). The functions are as follows: (1) The organic template participates in the formation of the process with the aluminosilicate and induces and regulates the construction of some pore structures. In addition, the zeolite desired structure can be obtained. (2) Template cations loaded into pores can balance the negative charge caused by the introduction of the framework aluminum atom, thus achieving the electroneutrality and stabilizing the crystal growth form. (3) Some organic templates can also change gel alkalinity, slow down the transformation of the unstable crystals into denser and stable structures, improving product stability, purity, and structure crystallinity. Although organics play an essential role in achieving accurate synthetic control of zeolites, its commercial utilization is plagued with many shortcomings: the use of organics increases the cost and costs as much as the material price as the other organics; to obtain open structures, templates must be burned at high temperatures for removal, which consumes energy and emits greenhouse gases; as many as dozens of organics may have harmful effects on both man and environment.

As a result, researchers gradually begin to use environmentally friendly alternative templates, which is mainly the use of biodegradable or low toxicity organic molecules and inorganic structure directing agents that completely replace the organic molecules to solve the problems caused by the use of organic molecules. They can complete the green and environment friendly synthesis of zeolite. For instance, Zhu et al. (2013) employed an environmentally friendly, inexpensive and non-toxic N-methylpyridinium iodide (NMPI) quaternary ammonium salt as a template to prepare the highly silica Y zeolite at 120 °C–130 °C crystallizing conditions. The material showed higher hydrothermal stability, hydrocarbon adsorption capacity and catalytic activity compared with commercial zeolites. Wang et al. (2009) synthesized AlPO-5 using tetramethylguanidine (TMG), which exists in the animal metabolites with lower toxicity, as a template, so this approach provides an efficient pathway for preparation of environment friendly AlPO-4 and its ordered derivatives. As TMG molecule contained four nitrogen atoms instead of only one atom present in the commonly used triethylamine, therefore coordination ability with Al is stronger, it was found that crystallization is very rapid when used as template. This way improved the yield of zeolite synthesis and provides a new thinking for the synthesis of green zeolite material. Ren et al. (2011) proposed a green and cheap strategy with the utilization of a copper amine complex (Cu-TEPA) as a template, they used the copper amine compound that formed a regular hexagonal channel crystal whose structure fits well with the CHA cage of SSZ-13 as template material and successfully prepared Cu-SSZ-13 which combined the functions of structural direction with introduction of copper into the framework, with great performance in ammonia selective catalytic reduction (NH_3_-SCR) reaction. Zones and Hwang (2002) adopted a newly synthesized SSZ-47, prepared polyamines rather than expensive templates from available and inexpensive raw material as structure directing agent. Liu et al. (2008) successfully synthesized an EMT-type zeolite using inexpensive polydimethyldiallylammonium chloride (a common polyquaternary ammonium salt used in shampoos and detergents) as a substitute for expensive 18-crown-6. The resulting material exhibited higher activity than Y type zeolites in cumene catalytic cracking reactions.

**FIGURE 3:**
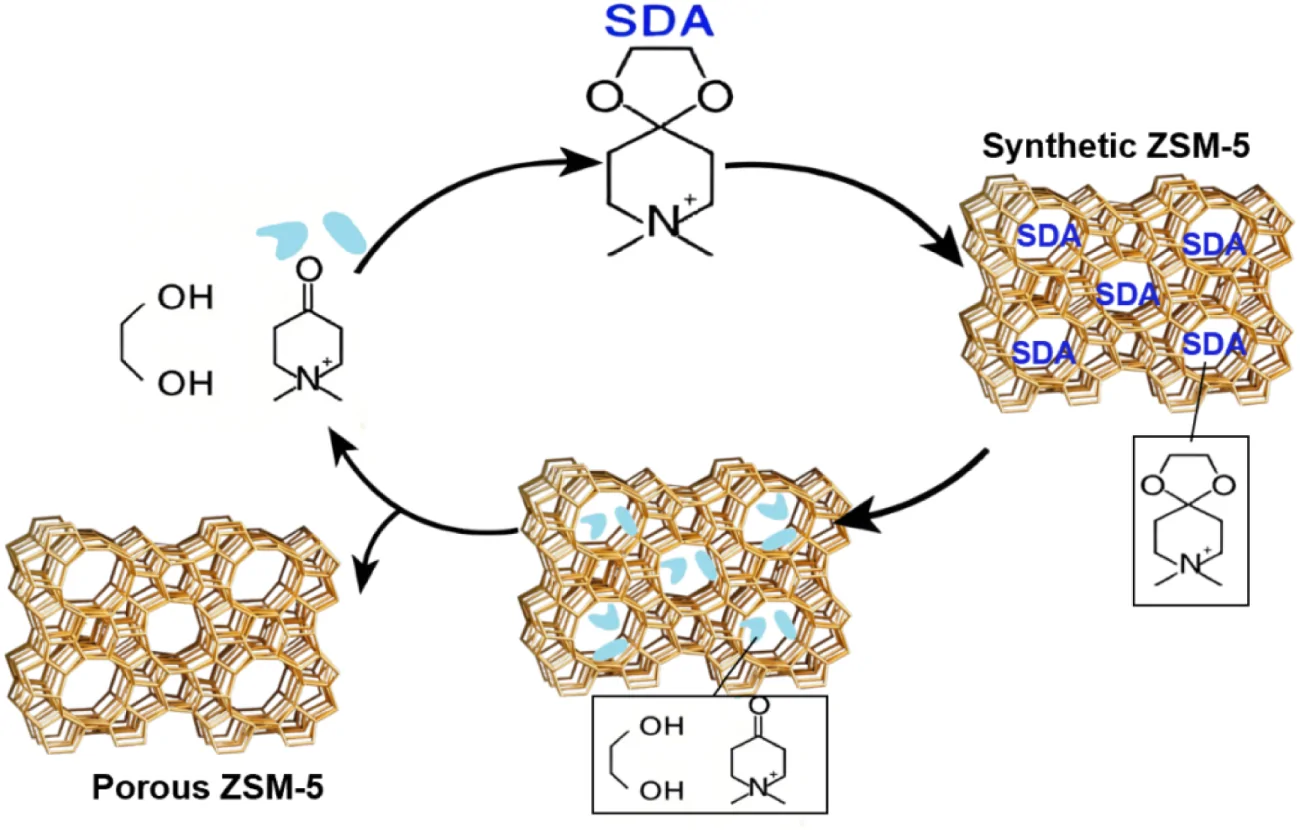
Schematic representations of synthesis of zeolites with recyclable organic templates synthetic methodology (redrawn based on Ref. (Lee et al., 2005)).

Beyond the development of greener OSDAs, template recovery and reuse provide another route to reduce the environmental and economic burden associated with conventional calcination-based removal. Lee et al. (2003) successfully extracted the triethanolamine template from a BEA* type zeolite using an acetic acid solution, leveraging the template’s small molecular size and weak interaction with the framework. The research group further proposed a “decomposition-recombination” strategy to realize template recycling. In this method, methyl iodide reacts with 1,4-dioxa-8-azaspiro [4.5] decane under strong alkaline conditions to form a template for synthesizing MFI-type zeolites. Under mildly acidic conditions, the template can be decomposed and separated from the framework without destroying the crystal structure, and the decomposition products can be recombined to regenerate the template. As illustrated in Figure 3, this strategy can also be extended to the synthesis of ZSM-11 and ZSM-12 zeolites (Lee et al., 2005). Compared with conventional template removal, it avoids high temperature calcination and significantly reduces environmental pollution, although these advantages come at the cost of increased reaction complexity and higher raw material consumption.

Overall, greener template design together with template recovery and reuse can reduce the environmental and economic burdens associated with conventional OSDAs while retaining their structure-directing function during zeolite crystallization.

### 2.2 Synthesis with green raw materials

The technology for synthesizing zeolites from traditional chemical raw materials such as sodium silicate and sodium aluminate is already very mature. The disadvantage of this method is that it heavily relies on these chemicals, resulting in high production costs. The manufacturing process of these raw materials itself is related to high energy consumption and environmental pollution. Developing alternative raw materials for economic and environmental protection has become a research focus in this field and is urgently needed. The dissolution behavior and chemical heterogeneity of renewable silica/alumina sources can influence precursor speciation and consequently affect nucleation kinetics and phase selectivity.

In terms of raw material innovation, research efforts have concentrated on the resource utilization of natural minerals and industrial waste. For example, due to its high silica content, rice husk ash has been widely used to synthesize nano Zeolite X (Akinjokun et al., 2024), NaA/NaX zeolite (Madhu et al., 2022a), and ZSM-5 zeolite (Ji F. et al., 2024; Klunk et al., 2021) under solvent free conditions or in synergy with kaolin. Flores et al. (2021) also synthesized a potassium zeolite from rice husk ash, demonstrating a pathway for the high value utilization of waste from the rice industry. Beyond rice husk ash, Tang et al. (2022) systematically investigated the sol gel behavior of kaolin and quartz under alkaline conditions, providing new insights for converting low cost mineral resources. Nguyen D. K. et al. (2023), Nguyen N. A. et al. (2023) synthesized microporous ZSM-5 zeolite from bentonite. Chen et al. (2021) directly synthesized magnesium doped ZSM-5 zeolite via a hydrothermal method using laponite and tetraethoxysilane as combined silica sources. Porous ZSM-5 and sodalite zeolites can also be synthesized from natural loess (Zheng et al., 2021) and other raw materials through relatively simple processes. Notably, industrial solid wastes like fly ash can serve as silicon and aluminum sources for the hydrothermal synthesis of zeolites after mechanical activation (Grabias-Blicharz et al., 2022). The successful use of fluidized catalytic cracking (FCC) residue (Guo et al., 2023) and biomass power plant ash (Liang et al., 2021) has further expanded the application scenarios for waste resources in zeolite synthesis. Parmar et al. (2025) and El Boaddayni et al. (2023) reviewed progress in synthesizing zeolites from natural or secondary resources such as kaolin, fly ash, and bauxite, highlighting the economic and environmental advantages of such precursors. Zhang et al. (2024) synthesized LTA zeolites from silicon-rich biomass ash via high-temperature silicon extraction followed by template-free hydrothermal crystallization. Madhu et al. (2022a) synthesized template-free NaA and NaX zeolites from rice husk ash-derived sodium silicate via a hydrothermal method, providing a sustainable route for valorizing agricultural waste. Representative studies on the synthesis of zeolites from environmentally friendly raw materials reported over the past five years are summarized in Table 2.

**TABLE 2:** Literature summaries have been available on the preparation of zeolites using environmentally friendly raw materials over the last 5 years, including their synthesis.

Category	Raw material/Process	Synthesized product	Key features/Advantages	Ref.
Raw Material Innovation	Rice Husk Ash	Nano Zeolite X and ZSM-5 Zeolite	High silica content, used under solvent free conditions or in synergy with kaolin	Akinjokun et al. (2024); Ji et al. (2024a); Klunk et al. (2021)
	Rice Husk Ash	NaA/NaX Zeolite	High silica content, enabling high value utilization of rice industry waste	Madhu et al. (2022a)
	Rice Husk Ash	Potassium Zeolite	Demonstrated a pathway for high value utilization of rice husk ash	Flores et al. (2021)
	Kaolin and Quartz	Zeolite	Systematically investigated sol gel behavior under alkaline conditions, providing insights for converting low cost mineral resources	Tang et al. (2022)
	Bentonite	Microporous ZSM-5 Zeolite	Synthesized from natural bentonite mineral	Nguyen et al. (2023a); Nguyen et al. (2023b)
	Fly Ash	Zeolite	Serves as silicon and aluminum source after mechanical activation for hydrothermal synthesis	Grabias-Blicharz et al. (2022)
	FCC Residue	Zeolite	Expanded application scenarios for waste resources in zeolite synthesis	Guo et al. (2023)
	Biomass Power Plant Ash	Zeolite	Expanded application scenarios for waste resources in zeolite synthesis	Liang et al. (2021)
Raw Material Innovation and synthesis Process Optimization	Laponite and Tetraethoxysilane	Mg-doped ZSM-5 Zeolite	Direct hydrothermal synthesis using combined silica sources. Imparts improved catalytic and adsorption properties	Chen et al. (2021)
	Natural Loess + Template free Method Zeolite	Porous ZSM-5 and Sodalite Zeolite	Synthesized through a relatively simple process	Zheng et al. (2021)

Although the aforementioned progress has significantly advanced the green synthesis of zeolites, further breakthroughs are still needed in the following directions: expanding the database of suitable low cost raw materials, particularly for the targeted conversion of region specific waste resources; and deepening the understanding of the synergistic mechanisms between solvents and templates to establish quantitative structure-process-performance relationships.

### 2.3 Template free synthesis

#### 2.3.1 Seed method

The seed assisted method is widely employed in the industrial synthesis of zeolite. By introducing seeds into the synthesis system, this approach can effectively shorten the induction period, accelerate crystallization, suppress the formation of impurity phases, and control crystal size. Research has demonstrated that a seed assisted, low alkalinity gel system can successfully synthesize MOR zeolite nano assemblies with a silicon-to-aluminum ratio (SAR) of 9.4 and a solid yield of 84%–94% under controlled, mild alkaline conditions (Cao et al., 2021). Furthermore, ZSM-5 zeolite has been synthesized via a seed assisted method using perlite tailings as the sole source of silicon and aluminum (Ren et al., 2024). Even in the absence of an organic structure directing agent (OSDA), the seed assisted method can be utilized to synthesize FER zeolite, where the crystallization time, synthesis cycle, and cost are significantly influenced by the physicochemical properties of the seed crystals (Patil et al., 2025). Additionally, hierarchical ZSM-5 zeolite with a mesoporous structure has been successfully prepared through a strategy combining boron doping with seed assistance, which exhibited excellent catalytic performance in heavy oil cracking reactions (Liu et al., 2022).

In the green synthesis, they also developed some ideas for synthesizing zeolites without using organic templates but seeds. For example, high crystallinity aluminosilicate-1 was prepared with adding mineralizer and solubilizing agent NH_4_F (NH_4_F: Si ratio of F^−^/Si is 0.25) and seeds (seeding with 5.6 wt%), it could stop amorphous silica formation to synthesize cheap kind of green zeolites (Meng et al., 2025). And another report demonstrated that ZSM-5 could be synthesized by a template free process just based on the usage of seeds with hierarchical pores (Shestakova et al., 2022). Seeds with a topological feature similar to framework density as well as identical structural unit could expedite the nucleation of ZSM-5 (Li et al., 2021). Even furtherly, a ZSM-5/beta composite zeolite from rice husk ash obtained with enhanced pore volume, catalytic activity and stability compared with that made under seed assisted method (Mirshafiee et al., 2022).

**FIGURE 4:**
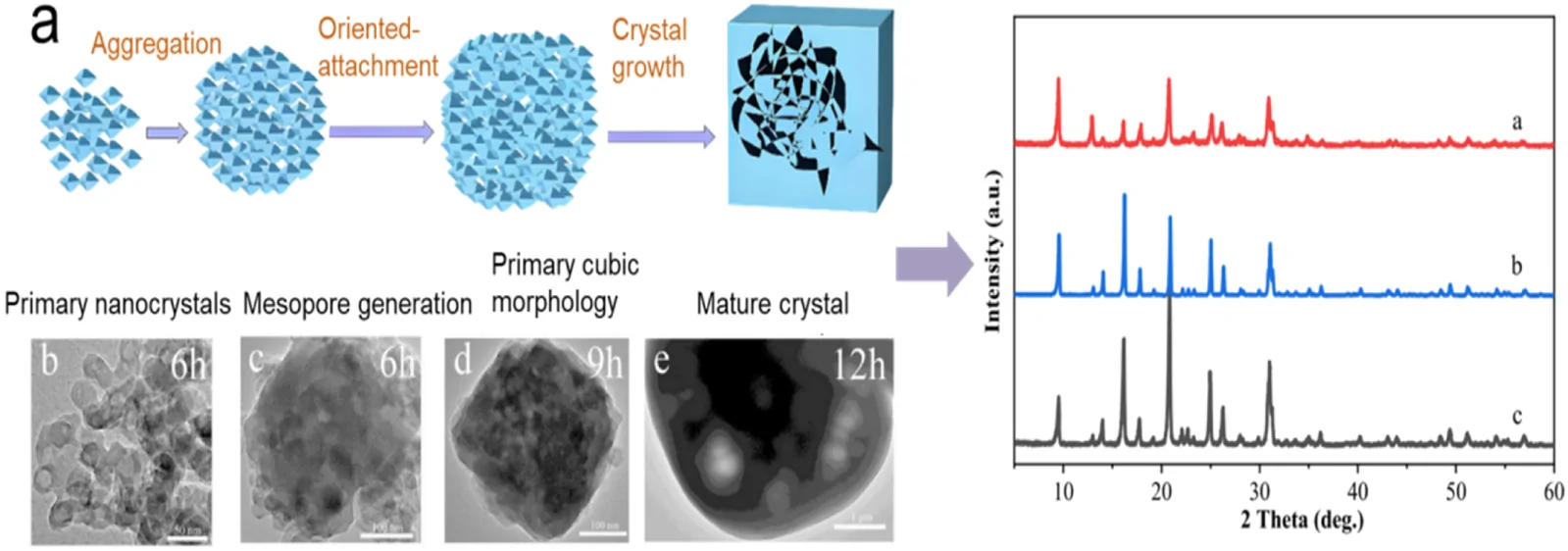
Schematic representation and **(b-e)** TEM images of the crystal growth process of SSZ-13-CA-S, XRD patterns of the SSZ-13-CA-S samples synthesized by adding CA and seed at **(a)** 6 h, **(b)** 9 h, **(c)** 12 h (redrawn based on Ref. (Li et al., 2022)).

In addition, the preparation of ECR-1 zeolite via a radical assisted seeding route is proposed in a way having good quality and rapidly. Furthermore, this zeolite can be served as potential catalysts with good performance (Lu et al., 2022). Also, we still have even MOR/ZSM-5 intergrown zeolite with seed assisted method without needing OSDA, and both zeolites showed well performance in toluene ethanol alkylation (Huang et al., 2023). Moreover, different zeolite frameworks including (MFI, TON, MTT, *MRE) are synthesized through the combined employment of zeolite seeds with alcohol filling strategy. Their resulted novel TF-Al-ZSM-5 material has improved MTP reactivity as well as catalytic stability toward MTP reaction (Luan et al., 2021). More importantly, porous layered mordenite can be synthesized by a seed assisted and template free way and this one displayed the same good catalytic performance towards ethanol dehydration reaction (Iadrat et al., 2021). Li et al. (2022) observed accelerated crystallization rates when seed crystals were added during the synthesis of SSZ-13 zeolite. They attributed this phenomenon to the addition of seed crystals promoting the formation of more secondary structural units in the initial gel during aging, thereby significantly accelerating the nucleation rate of the zeolite (Figure 4).

Besides above work, we can utilize seeding method design multifunctional material as well. In one case, directly using seed induced method could prepare nanosized zeolite with tunable acidity ZSM-5 without templates. After compositing with chromium oxide nanoparticles, the resulted multifunctional material was able to selectively convert CO to aromatics under hydrogenation (Xin et al., 2022). In another one, Nanozeolite ZSM-5 (NZSM-5) could be synthesized upon crystallization on surface nucleation nano ZSM-5. However, the silica-alumina zeolite-1 would be served as seed material with resulting nanozeolite has smaller particle size, large surface area, favorable catalytic performance and better catalytic stability than conventional NZSM-5 (Wang et al., 2025).

To summary, the special advantages of seeding method: (1) great extent shorten or even eliminate organic templates, so provides green synthetic method; (2) crystal growth time shortened considerably, greatly enhance its synthesis efficiency; (3) selected to crystallize desirable phase and suppressed undesirable one; (4) easily adjust size of the crystals; (5) easy to induce zeolite membranes for growing and structure various kinds of needed materials with required meso-/micro-structures, giving them bright perspective to application at industrial scale. Given these superior characteristics of zeolite synthesized by seeding method, this technology would be surely worthy of tremendous further investigations.

#### 2.3.2 Structure directing agent method

The directed agent method is a strategy for the directional synthesis of zeolites by introducing a liquid phase medium containing zeolite precursors or specific structural units (such as primary/secondary building units) into the synthesis system. The core mechanism of this method lies in utilizing preconstructed structural units in the liquid phase as crystallization templates to induce nucleation and crystal growth of the target zeolite.

Studies have shown that β zeolite with a low Si/Al ratio (SAR≈8-10) can be successfully prepared without an organic template by introducing trace TEA^+^ into the liquid phase and controlling the synthesis conditions (Zhou et al., 2000). MAS-5 zeolite with hexagonally symmetrical pores can be synthesized when an aluminosilicate precursor with a microporous mesoporous composite structure is present in the guiding solution. Spectral characterization confirmed that the material exhibits characteristic vibration peaks of five membered and six membered rings in the range of 400–600 cm^‒1^, indicating that zeolite like secondary building units are embedded in the pore walls, which impart high acid strength and excellent thermal stability (Zhang et al., 2001). TEM analysis further revealed that regular microporous sequences, transmitted via the guiding liquid, exist in zeolites such as MAS-7, MTS-9, and SBA-15, providing direct evidence for the templating effect of structural units in the liquid phase (Hasnan et al., 2020; Liu et al., 2002).

**FIGURE 5:**
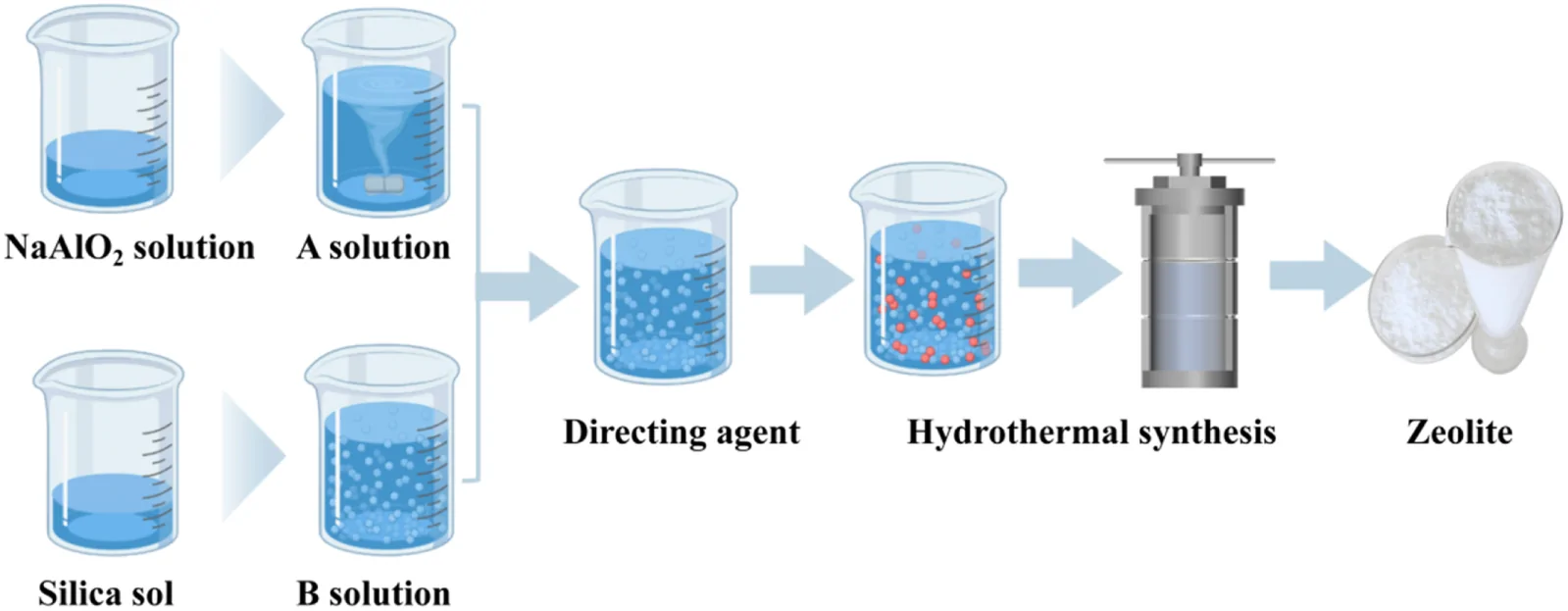
Flowchart of directing agent method synthesis.

Some studies have successfully induced the crystallization of ZSM-34 zeolite by using a guiding liquid containing secondary building units of L-type zeolite (such as the CAN cage) (Wu et al., 2008). In this process, the CAN cage in the guiding liquid acts as a topologically matched structural element, which effectively reduces the nucleation barrier of ZSM-34, thereby enabling its directional crystal growth without organic additives. A schematic illustration of the general directing-agent-assisted hydrothermal synthesis process is shown in Figure 5. Representative studies on the synthesis of zeolites using structure-directing agents reported over the past five years are summarized in Table 3.

**TABLE 3:** Literature summaries have been available on the preparation of zeolites using directing agent method synthesis.

Method/System	Target zeolite	Synthesis conditions/Characteristics	Performance/Structural features	Ref
Liquid phase medium with trace TEA^+^ introduction	Beta Zeolite	Organic template free, low Si/Al ratio (SAR≈8-10), controlled synthesis conditions	Successful synthesis of Beta zeolite with low Si/Al ratio and high crystallinity	Zhou et al. (2000)
Guided solution containing an aluminosilicate precursor with microporous-mesoporous composite structure	MAS-5	Presence of a microporous-mesoporous composite structure precursor in the guided solution	Hexagonally symmetrical pores; FTIR spectra show characteristic vibration peaks of five membered and six membered rings in the range 400–600 cm^‒1^, indicating zeolite like SBUs embedded in the pore walls, conferring high acid strength and excellent thermal stability	Zhang et al. (2001)
Transmission of structural units via the guiding liquid (confirmed by TEM analysis)	MAS-7, MTS-9, SBA-15	Templating effect provided by the guiding liquid	TEM reveals regular microporous sequences, providing direct evidence for the templating effect of structural units in the liquid phase, leading to the formation of ordered pore structures	Liu et al. (2002)
Using a guiding liquid containing secondary building units of L-type zeolite (e.g., CAN cage)	ZSM-34	Organic additive free, CAN cage acts as a topologically matched structural element, reducing the nucleation barrier	Induces directional crystallization of ZSM-34, enabling efficient crystal growth without organic templates	Wu et al. (2008)

Although the directed agent method can significantly enhance crystallization kinetics by shortening the crystallization period and suppressing impurity phases through the transfer of structural information its industrial application still faces several bottlenecks: (1) the preparation of the guiding liquid requires precise control over the hydrolysis and condensation processes of the precursor, increasing process complexity; (2) some systems still depend on auxiliary templates, making it difficult to achieve fully green synthesis; and (3) the stability and reproducibility of the directed agent have not been systematically addressed. Future research should focus on elucidating the self assembly mechanism of the guiding liquid and developing low energy preparation technologies to facilitate the large scale application of this method.

#### 2.3.3 Direct synthesis

The direct synthesis method refers to the preparation of zeolites with specific topological structure by adjusting the thermodynamic and kinetic conditions of the synthesis system without using organic templates. Because of its green and economic characteristics, this method has become an important research direction in the field of zeolite synthesis.

In the template free synthesis of Y-zeolite, the research shows that the competition between the crystallization of Y zeolite and P zeolite can be effectively controlled by adjusting the Na2O content and crystallization temperature of the initial gel in the sodium alkali system, so as to optimize the synthesis path and product purity (Pour and Mohammadi, 2021). NAA zeolites with cubic LTA structure were synthesized without template (Madhu et al., 2022b). Madhu et al. Successfully synthesized zeolites with different structures (SOD, LTA and Fau; Madhu et al., 2022c) through simple conventional hydrothermal crystallization method. The material showed good crystal structure and CO_2_ adsorption performance. NaX zeolite with hierarchical pore structure was successfully synthesized by low temperature activation of kaolin and *in situ* crystallization strategy, and showed good catalytic activity (Xiao et al., 2024).

For the synthesis of mordenite (MOR), sheet MOR crystals were successfully prepared by optimizing the high density gel system, which effectively improved the problem of mass transfer restriction (Lima et al., 2022). In addition, the template free intermittent hydrothermal method can be used to prepare high performance MOR zeolite membrane, which shows excellent separation efficiency in the process of isopropanol dehydration pervaporation (Li et al., 2024). Template free direct synthesis has significant advantages in environment and economy. For example, it is used to prepare platinum containing MOR catalyst and has excellent performance in ethylbenzene isomerization (Kireev et al., 2023).

In terms of green synthesis of ZSM-5 zeolite, the selection of silicon and aluminum sources has an important impact on the crystallization behavior. For example, sodium metaaluminate helps to form ZSM-5 with high crystallinity, while aluminum nitrate easily leads to amorphous heterophases (Bragina et al., 2024). By adjusting the synthesis conditions, ZSM-5 with high silicon aluminum ratio can also be prepared (Cheng et al., 2023). In addition, the introduction of seed assisted method can realize the rapid synthesis of H-ZSM-5 within 1 h, significantly reducing energy consumption and environmental pollution (Tian et al., 2021). The developed template free solid state crystallization technology realizes the large scale production of ZSM-5 through the mechanical mixing process, with simple process and low cost (Yin et al., 2024). Small grain Y zeolite (Meng et al., 2021) and mesoporous materials with ZSM-5 topology (Demikhova et al., 2023) were successfully synthesized from cheap raw materials, showing good catalytic and adsorption properties (Jia et al., 2021). ZSM-5 zeolite synthesized directly without template shows high reactivity in n-heptane cracking due to its adjustable pore structure and acidity (Pour and Mohammadi, 2022). The template free synthesized ZSM-5 zeolite catalyst with atomically dispersed Fe-Cu sites enables the oxidative carbonylation of methane (Cheng et al., 2024).

For the preparation of CHA zeolite, a template free zeolite conversion method was developed to synthesize Cu-CHA catalyst, which showed excellent oxidation activation ability in NH_3_-SCR reaction due to its rich isolated aluminum sites (Lv et al., 2022). Alkali metal fluorides (such as NaF, KF, etc.) were found to promote the formation of dense ZSM-5 zeolite membranes (Si et al., 2021). In addition, nano-sized CHA zeolite was successfully synthesized by colloidal suspension method, which systematically revealed the influence of cation pairing effect on the crystallization path (Ghojavand et al., 2021).

In the synthesis of other types of zeolites, a variety of direct synthesis strategies without templates have emerged. For example, hydrophobic β zeolite can be prepared by combining *in situ* silicon source modification technology to improve the synthesis efficiency of lactic anhydride (Ma et al., 2025); Tin free aluminosilicate CHA zeolite and its metal modified catalyst can be synthesized in fluoride medium (Nasser et al., 2021); Aluminum rich ZSM-5 zeolite membrane can also be prepared on α- Al_2_O_3_ carrier by adjusting si/al ratio and fluorine salt type (Si et al., 2021). In terms of catalytic application, LSX zeolite with high crystallinity can be directly synthesized without aging and strong alkali assistance to improve the hydrogenation performance of CO_2_ (Xiang et al., 2023) Representative studies on the direct synthesis of zeolites without templates reported over the past five years are summarized in Table 4.

**TABLE 4:** Literature summaries have been available on the preparation of zeolites using direct synthesis over the last 5 years.

Method	Target zeolite	Key findings/Performance	Ref.
Adjusting Na_2_O content and crystallization temperature	Y-Zeolite	Optimized synthesis path, high product purity	Pour and Mohammadi, (2021)
Template free synthesis	NAA (LTA structure)	Successfully synthesized cubic LTA zeolite	Madhu et al., (2022b)
Conventional hydrothermal method	SOD, LTA, Fau	Good crystal structure, good CO_2_ adsorption performance	Madhu et al., (2022c)
Low temperature activation and *in situ* crystallization	NaX (Hierarchical)	Hierarchical pore structure, good catalytic activity	Xiao et al., (2024)
Optimizing high density gel system	Mordentie (MOR)	Sheet like crystals, improved mass transfer limitations	Lima et al., (2022)
Intermittent hydrothermal method	MOR Zeolite Membrane	Excellent separation efficiency for isopropanol dehydration via pervaporation	Li et al., (2024)
Template free direct synthesis	Pt-MOR Catalyst	Excellent performance in ethylbenzene isomerization; significant economic and environmental advantages	Kireev et al., (2023)
Selection of Al source (NaAlO_2_ vs. Al(NO_3_)_3_)	ZSM-5	Sodium aluminate promotes high crystallinity; aluminum nitrate leads to amorphous phases	Bragina et al., (2024)
Adjusting synthesis conditions	High Silica ZSM-5	Significant performance in selective oxidation of methane	Cheng et al., (2023)
Seed assisted method	H-ZSM-5	Rapid synthesis (<1 h), reduced energy consumption and environmental impact	Tian et al., (2021)
Solid state crystallization	ZSM-5	Simple process, low cost, suitable for large scale production	Yin et al., (2024)
Using cheap raw materials	Small crystal Y Zeolite	Good catalytic and adsorption properties	Meng et al., (2021)
Using cheap raw materials	Mesoporous material with ZSM-5 topology	Good catalytic and adsorption properties	Demikhova et al., (2023)
Template free direct synthesis	ZSM-5	High reactivity in n-heptane cracking due to adjustable pore structure and acidity	Pour and Mohammadi, (2022)
Template free synthesis	ZSM-5 with atomically dispersed Fe-Cu sites	Enables oxidative carbonylation of methane	Cheng et al., (2024)
Zeolite conversion method	Cu-CHA Catalyst	Excellent activity in NH_3_-SCR reaction due to isolated Al sites	Lv et al., (2022)
Using Alkali Metal Fluorides (NaF, KF)	Dense ZSM-5 Zeolite Membrane	Promotes formation of dense membranes	Si et al., (2021)
Colloidal suspension method	Nano-sized CHA Zeolite	Revealed influence of cation pairing effect on crystallization path	Ghojavand et al., (2021)
*In situ* silicon source modification	Hydrophobic β Zeolite	Improved synthesis efficiency for lactic anhydride	Ma et al., (2025)
Fluoride medium synthesis	Sn-free Aluminosilicate CHA and metal-modified catalysts	Successfully synthesized specified catalysts	Nasser et al., (2021)

Template-free direct synthesis provides a promising route for reducing the environmental and economic burdens associated with conventional organic templates. However, broader implementation remains constrained by challenges in controlling nucleation, crystal growth, phase selectivity, and framework diversity. Further mechanistic understanding and the development of more generalizable synthesis routes are therefore required to improve reproducibility and facilitate scale-up.

Although these green hydrothermal strategies have significantly improved the sustainability of zeolite synthesis, optimization of precursor composition, template dosage, alkalinity, crystallization temperature, and reaction time still relies heavily on empirical trial-and-error approaches. Machine learning offers a promising means of establishing quantitative relationships between synthesis parameters and crystallization outcomes, thereby facilitating the optimization of sustainable hydrothermal synthesis. The experimental data generated from these green synthesis strategies can therefore provide an important basis for the AI-assisted synthesis optimization discussed in Section 7.

Overall, hydrothermal synthesis remains the most mature and versatile route for zeolite preparation, while recent advances in greener templates, renewable raw materials, and template-free synthesis have substantially reduced its environmental burden. Nevertheless, achieving simultaneous control over crystallization efficiency, phase selectivity, structural diversity, reproducibility, and sustainability remains a key challenge for further scale-up and industrial implementation.

## 3 Ionothermal synthesis

As an innovative strategy, ionothermal synthesis utilizes ionic liquids or deep eutectic solvents (DES) as reaction media to enable controlled zeolite crystallization under low volatility conditions, providing a novel platform for developing new zeolites and studying crystallization mechanisms. In ionothermal synthesis, ionic liquids modify the solvation environment and interactions among inorganic precursor species, thereby influencing nucleation, framework assembly, and phase selectivity.

**FIGURE 6:**
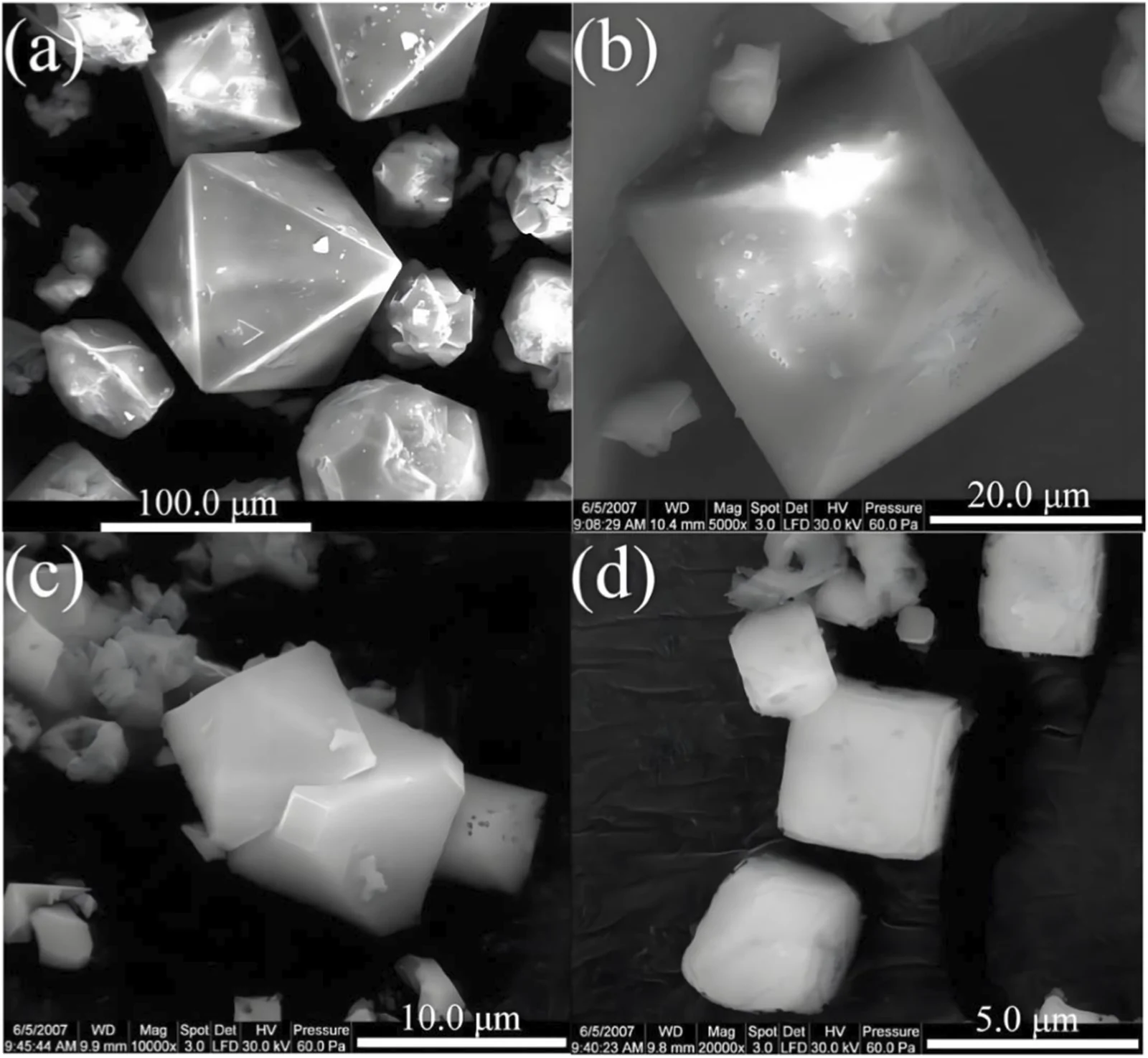
SEM images of **(a)** LTA-C3, **(b)** LTA-C4, **(c)** LTA-C5 and **(d)** LTA-C6 (redrawn based on Ref. (Ma et al., 2009)).

Ionothermal synthesis has proven to be an effective route for the preparation of large zeolite single crystals, and the physicochemical properties of ionic liquids, particularly the alkyl chain length of their cations, play a crucial role in determining crystal size and morphology. During the synthesis of GaPO_4_ zeolites, Ma et al. (2009) observed that increasing the alkyl chain length of 1-alkyl-3-methylimidazolium bromide ionic liquids (ILs) from C_2_ to C_6_ led to a significant reduction in the crystal size of GaPO_4_-LTA and a concomitant morphology transformation from octahedral to cubic crystals (Figure 6). These results demonstrate a strong correlation between the alkyl chain length of the ionic liquid cation and the resulting crystal size and morphology. Further mechanistic investigations revealed that fluoride ions (F^−^) play a decisive role in phase selection during ionothermal synthesis. Nuclear magnetic resonance (NMR) analyses showed that the presence of F^−^ favors the formation of GaPO_4_-LTA and GaPO_4_-CLO structures, whereas the fluoride free system yields the GaPO_4_-α phase. Conventionally, F^−^ is introduced through highly toxic hydrofluoric acid (HF). Although ammonium fluoride ([NH_4_]F) represents a safer alternative, it generally requires higher synthesis temperatures and larger dosages than HF. More complex fluoride containing compounds, such as tetramethylammonium fluoride ([Me_4_N]F), have also been employed as mineralizing agents in the ionothermal synthesis of LTA-type zeolites (Azim et al., 2018). These findings indicate that the concentration and nature of fluoride species strongly influence product structure and phase selectivity, providing a straightforward strategy for tailoring zeolite microstructures. The multifunctional role of F^−^ in ionothermal synthesis has been widely recognized for both aluminosilicate (Camblor et al., 1999; Zones et al., 2005) and aluminophosphate zeolites (Morris et al., 2004). In addition to enhancing the dissolution of precursor species through complexation effects (Xu et al., 2013), F^−^ can act cooperatively with ionic liquids as a structure directing agent (Bull et al., 2000; Morris, 2005).

Specifically, the incorporation of fluoride ions introduces negative charges into the zeolite framework, thereby strengthening electrostatic interactions between the framework and the ionic liquid cations. The strength of these interactions is governed by the charge density of the ionic liquid cation, which increases as the alkyl substituent becomes shorter. Consequently, ionic liquids with shorter alkyl chains exhibit stronger framework cation interactions, leading to enhanced stabilization of the growing framework and facilitating the formation of large single crystals. Besides the cation effect, the structure directing behavior of ionic liquids is also highly dependent on the nature of their anions. Different ionic liquid anions can result in distinct crystallization pathways and framework structures (Azim et al., 2018). Parnham and Morris (2006a) demonstrated that replacing [Emim]Br with [Emim][TF_2_N] altered the crystallization outcome from a zeolitic framework to chain structured Al(H_2_PO_4_)_2_F. This phenomenon was attributed to the lower polarity of [Emim][TF_2_N], which generates a heterogeneous distribution of polar and nonpolar domains within the reaction medium, thereby modifying crystallization kinetics and ultimately directing the formation of a different crystal structure.

**FIGURE 7:**
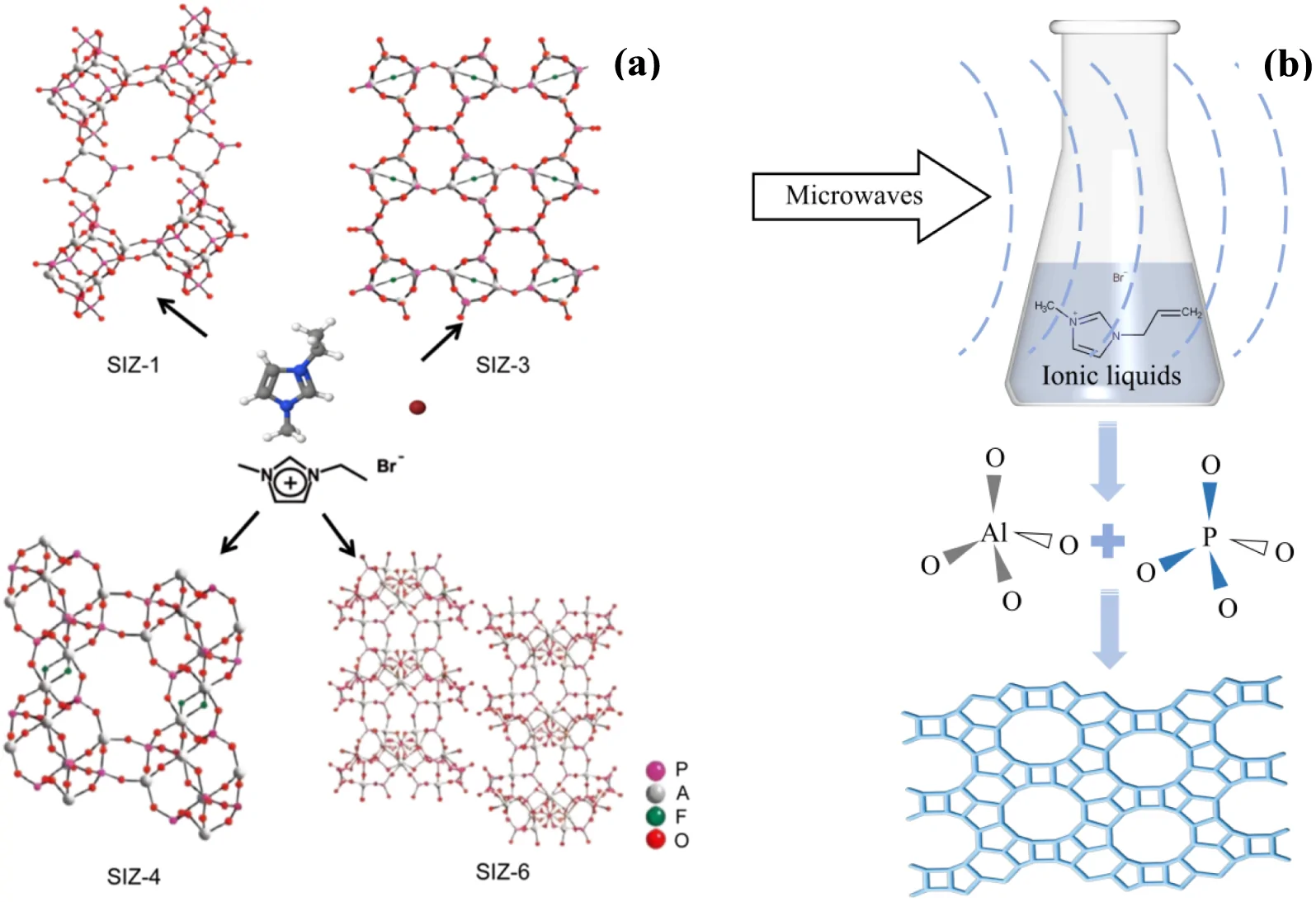
Aluminophosphate materials previously synthesized ionothermally from EMIBr **(a)**, (redrawn based on Ref. (Parnham and Morris, 2006c)) Simplified diagram of the microwave enhanced ionothermal synthesisof zeolites **(b)** (redrawn based on Ref. (Xu et al., 2006)).

When researchers successfully prepared the aluminophosphate zeolite SIZ-1 with a unique topology by using 1-ethyl-3-methylimidazolium bromide ([EMIM]Br) ionic liquid as both solvent and structure directing agent, while adjusting the ratio of aluminum isopropoxide to phosphoric acid (Cooper et al., 2004). Subsequent studies demonstrated that cobalt doped aluminophosphate materials (CoAlPOs) synthesized via a similar strategy exhibited significant advantages in catalysis and gas adsorption due to the redox activity of Co^2+^ ions (Parnham and Morris, 2006b). The designability of ionic liquids enables the regulation of zeolite pore structures and surface properties through cation/anion combinations, facilitating the synthesis of a series of functional zeolites (Ma et al., 2008; Morris, 2009; Parnham and Morris, 2006a; Parnham and Morris, 2007; Wheatley et al., 2010). When 1-ethyl-3-methylimidazolium bromide (C_2_ chain) ionic liquid was used as the template, SIZ-4 or SIZ-6 structures were obtained, whereas extending the alkyl chain to C_3_-C_4_ led to the formation of AlPO frameworks with the same topology as SIZ-4 only in the presence of HF (Parnham and Morris, 2006c; Figure 7a).

Ionic liquids are particularly attractive media for microwave assisted synthesis because of their excellent microwave absorption capability, negligible vapor pressure, and high thermal stability. Their resistance to decomposition into volatile species at elevated temperatures enables reactions to be performed under low pressure or ambient pressure conditions, providing a promising platform for zeolite synthesis. Moreover, the synergistic interaction between ionic liquids and microwave irradiation can effectively eliminate the temperature gradients commonly encountered in conventional heating processes. The resulting homogeneous and rapid heating facilitates precursor dissolution and mass transport, thereby promoting nucleation and accelerating zeolite crystallization kinetics. Building upon these advantages, Wang et al. (2006), (Xu et al., 2006) were the first to introduce microwave heating into an ionothermal synthesis system (Figure 7b). Under ambient pressure conditions, highly crystalline AEL-type zeolite crystals with controllable morphologies were synthesized within only a few minutes, demonstrating a dramatic reduction in crystallization time compared with conventional ionothermal methods. This work highlighted the potential of microwave assisted ionothermal synthesis as an efficient strategy for the rapid preparation of zeolite materials while maintaining excellent crystallinity and morphological control.

Compared to conventional solvothermal methods, ionothermal synthesis offers several core advantages: (1) the very low vapor pressure of the ionic medium significantly reduces the reaction system pressure and improves operational safety; (2) a wide liquid temperature range (−50 °C–400 °C) and high thermal stability enable precise control over high temperature crystallization processes; (3) the tunability of ionic liquids provides chemical space for the directional design of zeolites, for instance, by inducing the assembly of specific secondary building units through cation/anion selection; and (4) green synthesis characteristics, as most ionic liquids are water soluble and recyclable, avoiding the environmental risks associated with volatile organic solvents. Current research focuses on elucidating the interaction mechanisms between ionic liquids and zeolite precursors, particularly the spatial confinement effects and charge matching principles of ion pairs during nucleation. By integrating computational simulations with *in situ* characterization techniques, it is anticipated that structure activity relationship models for ionothermal synthesis will be established, advancing the rational design and the rational design and scalable synthesis of zeolites. Recent advances in machine learning also provide opportunities for optimizing ionic liquid selection, crystallization conditions, and framework prediction, thus improving the efficiency of ionothermal synthesis. Compared with conventional hydrothermal synthesis, ionothermal synthesis offers lower vapor pressure, improved crystallization control, and access to unique framework structures. Nevertheless, the relatively high cost of ionic liquids, difficulties in solvent recovery, and limited industrial experience currently restrict its large-scale application.

## 4 Dry gel conversion synthesis

**FIGURE 8:**
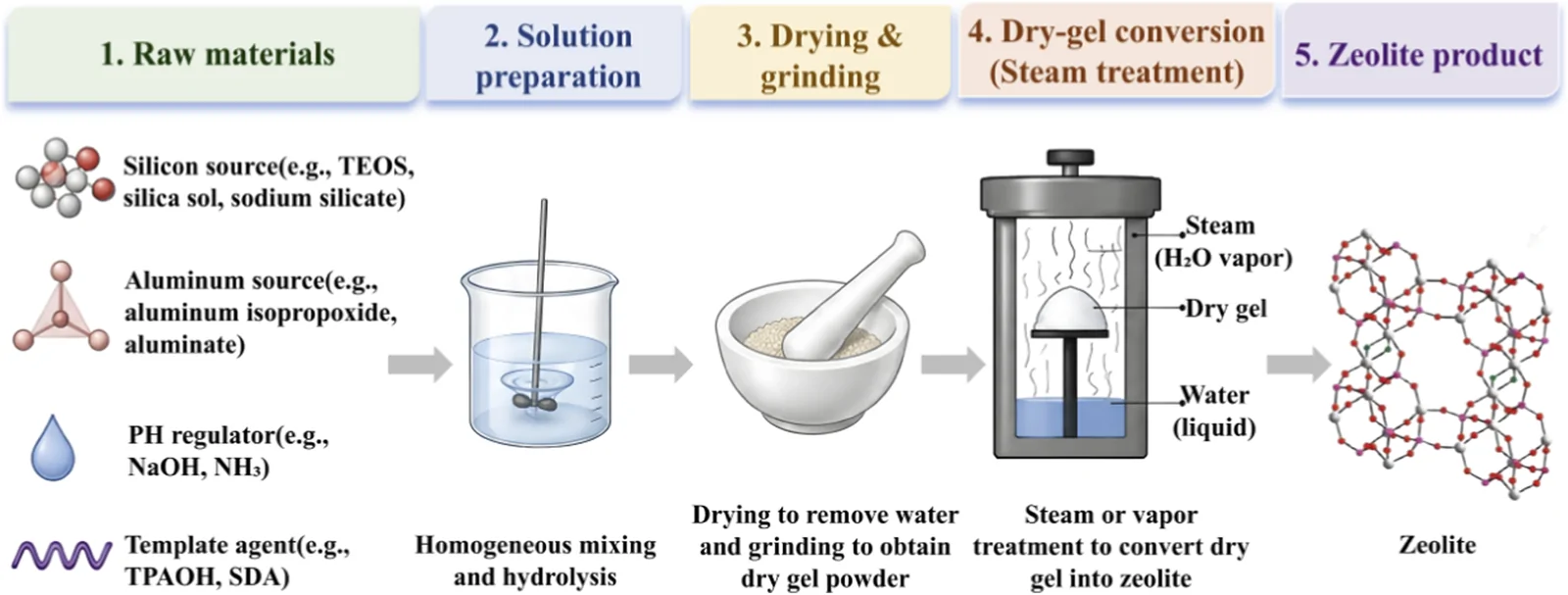
Flow diagram of dry-gel conversion.

Dry gel conversion (DGC) has attracted considerable attention as an alternative strategy for zeolite synthesis. In contrast to conventional hydrothermal synthesis, DGC first converts the precursor mixture into a dry gel, which is subsequently crystallized in the presence of water vapor, as illustrated in Figure 8. Because the dry precursor is physically separated from the liquid water during crystallization, DGC can substantially reduce bulk solvent consumption while maintaining the water required for hydrolysis, condensation, and framework formation. This approach can therefore reduce wastewater generation and, under appropriate conditions, facilitate zeolite crystallization with relatively low solvent demand.

In DGC, the limited liquid phase and vapor-mediated transport create a crystallization environment distinct from that of conventional hydrothermal synthesis. Water vapor promotes precursor mobility and hydrolysis–condensation while maintaining relatively low bulk solvent consumption, making water activity and vapor transport critical parameters governing nucleation and crystal growth. Consequently, the amount of water, vapor transport, temperature, and precursor composition can strongly influence crystallization kinetics, phase selectivity, crystal size, and morphology.

DGC has been applied to the synthesis of zeolites with different framework structures and crystal morphologies (Kim et al., 2025), while early studies demonstrated the feasibility of vapor-assisted conversion of dry precursors for zeolite crystallization (Xu et al., 1990). The general synthesis procedure of DGC is schematically illustrated in Figure 8.

MFI zeolite is one of the most popular zeolites, while DGC has unique characteristics in its crystallization process. For example, crystallization of MFI zeolite could be easily achieved and controlled by adjusting the addition amounts of tetrapropylammonium bromide (TPABr) and NaOH, e.g., molar ratio of NaOH/SiO_2_ = 0.05625 and TPABr/SiO_2_ = 0.05625.; meanwhile, introducing carbon black as hard templates in MFI zeolite successfully forms mesoporous crystal, which is otherwise difficult to synthesize using the hydrothermal method because both the template and the crystal structure tend to separate (Kim et al., 2025). In addition, a new ammonia assisted DGC was presented, dramatically enhancing the crystallization and catalytic performance of MFI zeolite (Lin et al., 2025). While developing catalysts with other structures such as hierarchical microstructure using DGC, we prepared the shape selective catalyst based on Pt@MFI using DGC by loading NPs of Pt, and then coating the zeolite shell on it (Endoh et al., 2024). TS-1 is highly important for selective oxidation reactions, and the dry gel conversion (DGC) method can effectively enhance the incorporation efficiency of titanium species into the framework and improve its catalytic performance. The ammonia assisted DGC strategy promotes the formation of Ti(OSi)_3_OH active sites, thereby enhancing H_2_O_2_ activation capability and oxidation activity (Lin et al., 2025). Studies have also demonstrated that this method can be used to synthesize nanoscale, monolithic TS-1 crystals and clearly reveal their crystallization pathway and structure catalytic performance relationship (Ji et al., 2024b; Ji et al., 2024c).

The DGC method is also suitable for the green synthesis of BEA-structured zeolites. Using silica extracted from rice husk ash as the silicon source, BEA/Fe_3_O_4_ composites were successfully prepared, enabling the high value utilization of waste resources; these composites exhibit excellent adsorption performance for heavy metal ions in water (Phouthavong et al., 2024). In another study, using silicon fluoride slag as a raw material, a Mg-modified Hβ zeolite was synthesized via a tetraethylammonium hydroxide (TEAOH) directed DGC process, demonstrating good catalytic potential (Wang and Zhao, 2024). The DGC method has broad applicability and can also be employed to synthesize various structures, including sodalite (Liu X. S. et al., 2025) and Fe-ZSM-5 (Luan et al., 2024). For instance, RHO zeolite was synthesized via steam assisted conversion (SAC). Systematic investigation revealed that the optimal synthesis conditions involve reaction at 140 °C for 48 h with 0.5 mL of water per 1 g of dry gel. Under these conditions, high purity, highly crystalline RHO zeolite can be obtained. Auxiliary methods such as ultrasonic treatment also help optimize its crystalline phase composition and particle size (Brazovskaya et al., 2025). Fe-ZSM-5 with hierarchical porosity can be prepared using an Fe-MOF precursor. Furthermore, employing an amphiphilic silane as a mesoporous template combined with a metal impregnation strategy enables the construction of Mn-loaded hierarchical Fe-ZSM-5 catalysts (Diao et al., 2024). Seed assisted DGC (SA-DGC) has also been used for the efficient synthesis of highly crystalline nano sized ZSM-5, significantly increasing yield and reducing crystallization time (Gou et al., 2025). This approach can be applied to synthesize high performance mordenite (MOR) membranes, which exhibit excellent separation performance and stability on macroporous alumina supports (Wang et al., 2022).

In summary, dry-gel conversion (DGC) is a promising green synthesis route that substantially reduces bulk solvent consumption while enabling effective control over zeolite crystallization and structural characteristics. Its limited liquid and vapor-mediated crystallization environment provides opportunities for framework and morphology control, hierarchical structure formation, and the utilization of alternative raw materials. Nevertheless, DGC is highly sensitive to water activity, humidity distribution, gel homogeneity, and heat and mass transfer, which may become increasingly difficult to control during scale-up. Future research should therefore focus on elucidating vapor-mediated crystallization mechanisms, improving process reproducibility, and developing continuous or semi-continuous reactor configurations. In this context, machine-learning-assisted process optimization may further facilitate the identification of suitable operating windows and the control of humidity, crystallization conditions, and product quality, thereby supporting the scale-up of DGC.

## 5 Electrochemical deposition

Electrochemical deposition offers a novel approach for the rapid preparation of zeolite coatings. The applied electric field can alter the local ionic environment and interfacial transport near the electrode surface, providing an additional means of regulating heterogeneous nucleation and subsequent zeolite growth.Recent studies demonstrate that cathode electrodeposition enables controllable growth of zeolitic phases within 30 min. This technique features the following advantages: (1) low energy consumption as it operates without high temperature or pressure; (2) precise control over crystal polymorphism via potential regulation; and (3) environmental friendliness by avoiding organic solvents. This technology opens a green pathway for the scalable production of functionalized zeolite coatings (Warty et al., 2024).

External electric fields have provided unprecedented opportunities for regulating zeolite synthesis and electrochemical synthesis processes. However, the incorporation of electric fields into synthesis systems still faces several practical challenges, including the requirement for sealed reaction environments and the narrow electrochemical window of aqueous systems. Ionic liquids, characterized by wide electrochemical windows, high ionic conductivity, and negligible vapor pressure, offer a promising alternative for applying electric fields under ambient pressure conditions. Based on these advantages, Cai et al. (Yu et al., 2015) first proposed a one step *in situ* electrochemical synthesis strategy by introducing an external electric field into an ionothermal system. By applying a constant negative potential, an AlPO_4_-11 zeolite coating with excellent corrosion resistance was successfully deposited on an aluminum cathode substrate. Notably, the coating formed on the cathode rather than the anode. Its formation mechanism was mainly attributed to electrostatic interactions: the negatively charged cathode surface attracts cations from the ionic liquid, thereby inducing the formation of the zeolite framework, whereas the anode electrostatically repels cations and is therefore unfavorable for zeolite growth. This method establishes a simple, controllable synthesis system that can operate under ambient pressure conditions, demonstrating significant potential for industrial applications and for investigating the growth mechanisms of zeolite films.

**FIGURE 9:**
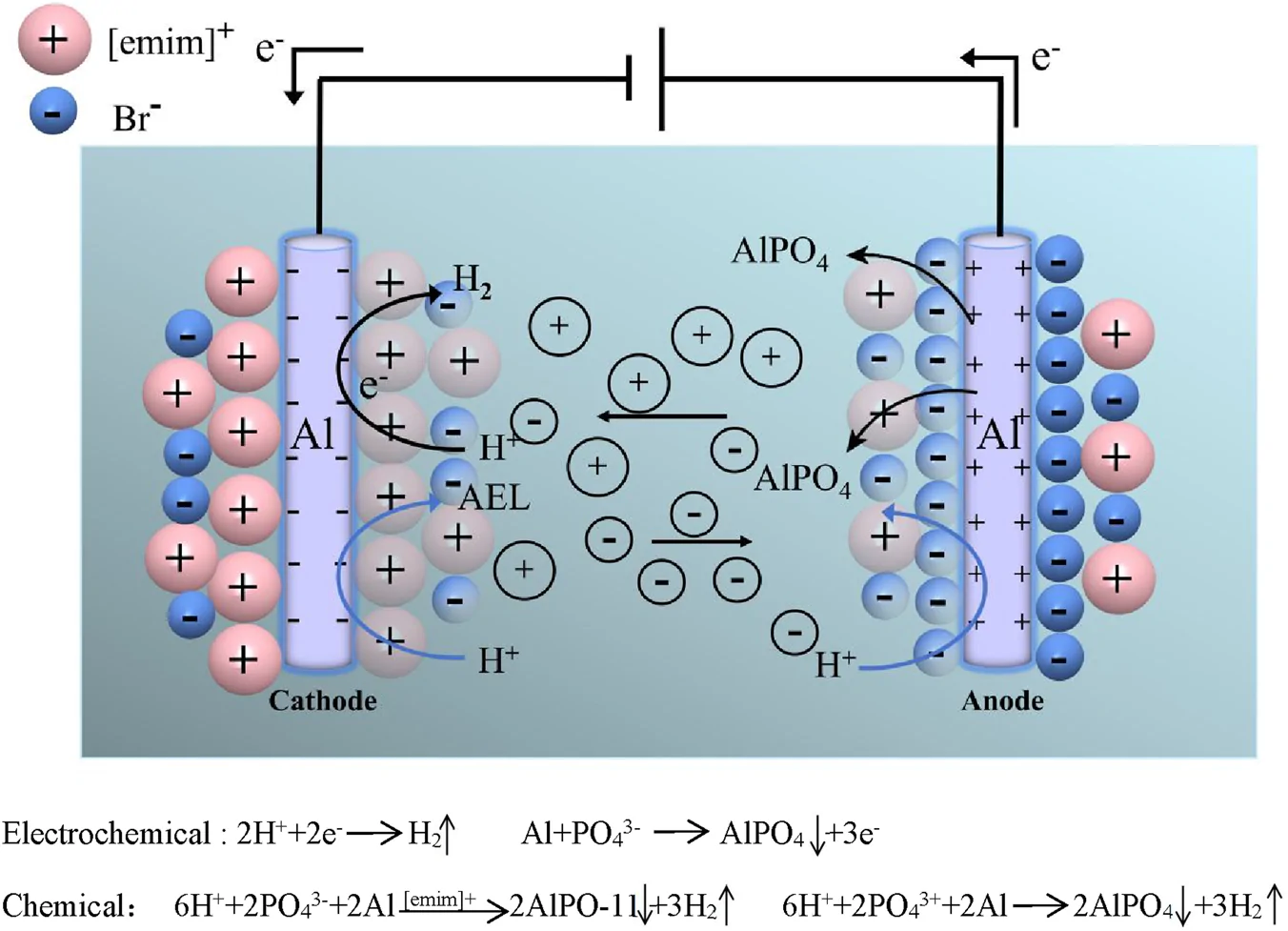
Schematic Illustration of the proposed in-situ electrochemical ionothermal synthesis mechanism of the AlPO_4_-11 film (redrawn based on Ref. (Yu et al., 2017)).

In subsequent studies, alternating positive and negative currents were employed to achieve both the electrochemical oxidation of the aluminum substrate and the conversion of aluminum species into the zeolite framework (Figure 9; Yu et al., 2017). Each cycle consisted of a positive current stage followed by a negative current stage, facilitating nucleation. By regulating the number of cycles and the current density at low temperatures, the microstructural evolution of the seed layer could be precisely controlled, including the formation of amorphous gels and the embedding of crystal seeds within the gel matrix. Subsequently, the application of a constant negative potential at elevated temperatures induced the epitaxial growth of zeolite films with in plane orientation (Han et al., 2023).

The transition from conventional hydrothermal synthesis toward more sustainable zeolite production relies on the complementary development of multiple green synthesis strategies rather than on a single technological solution. Each strategy addresses different aspects of resource consumption, environmental impact, and crystallization control. For example, template-free synthesis reduces the dependence on organic structure-directing agents, dry-gel conversion substantially decreases bulk solvent consumption, ionothermal synthesis provides alternative reaction media and distinct crystallization environments, and electrochemical synthesis enables electrically controlled crystallization under relatively mild conditions. However, no single strategy simultaneously achieves low energy and solvent consumption, minimal waste generation, precise crystallization control, and straightforward industrial scale-up.

Table 5 summarizes representative green zeolite synthesis strategies in terms of solvent usage, template consumption, energy demand, waste generation, and industrial scalability. This comparison provides a holistic perspective for selecting appropriate synthesis approaches and establishes a foundation for the subsequent discussion of machine-learning-assisted optimization and intelligent design. The synthesis strategies described above also generate experimental data linking precursor composition, synthesis conditions, and crystallization outcomes, which can be integrated with machine-learning models for reaction optimization, process control, and intelligent synthesis design. Electrochemical synthesis provides precise control over nucleation and crystal growth under mild conditions, but its productivity, electrode stability, and reactor scalability require further investigation before large-scale industrial implementation.

**TABLE 5:** Comparison of green zeolite synthesis strategies.

Method	Main synthesis mechanism	Solvent usage	Template usage	Energy demand	Waste generation	Major advantages	Major limitations	Key challenges
Conventional hydrothermal	Dissolution–recrystallization involving precursor dissolution, nucleation, and hydrothermal crystal growth	High	High	High	High	Mature process; broad framework applicability; well-established synthesis control	High water and OSDA consumption; alkaline wastewater; energy-intensive heating and post-calcination	Reduce water, energy and OSDA consumption; improve template recovery
Green template	Structure-directed crystallization using lower-toxicity, recyclable, or more resource-efficient templates	Medium	Medium	Medium	Medium	Retains structure-directing capability while reducing environmental burden	Template synthesis/recovery is still required; additional cost may remain	Develop inexpensive, recyclable and highly efficient green templates
Template free	Seed-induced or inorganic-species-directed nucleation and framework growth without OSDAs	Low	Low	Medium	Low	Avoids costly OSDAs and template-removal calcination; environmentally favorable	Limited accessible framework types; narrower crystallization windows	Expand topology diversity and improve reproducibility
Ionothermal	Ionic-liquid-mediated precursor dissolution, nucleation and framework crystallization	Very Low	Low	Medium	Low	Negligible vapor pressure; mild pressure; access to unusual frameworks	High ionic-liquid cost; recovery and purification requirements	Ionic-liquid recycling, cost reduction and reactor/process optimization
Dry gel conversion	Vapor-assisted conversion of a preformed dry gel through limited-water transport and crystallization	Very Low	Medium	Low	Low	Low solvent demand; reduced wastewater; efficient precursor utilization	Sensitive to gel homogeneity, humidity and vapor transport	Uniform heat/mass transfer and continuous processing
Electrochemical deposition	Electric-field/electrochemically controlled nucleation and crystal growth	Low	Low	Low	Low	Mild conditions; spatial and temporal control of nucleation/crystal growth	Low productivity; electrode and reactor limitations	Reactor scale-up, electrode durability and pilot-scale validation

## 6 Industrialization perspectives of green zeolite synthesis

Although numerous green synthesis strategies have demonstrated significant environmental advantages at the laboratory scale, their practical implementation depends on economic feasibility, process robustness, and industrial scalability. Beyond improving synthesis efficiency and reducing environmental impacts, successful industrialization requires balancing raw material costs, energy consumption, equipment investment, product quality, and process stability. Therefore, techno-economic evaluation and scale-up considerations have become indispensable for translating laboratory-scale green synthesis into commercial production.

### 6.1 Techno-economic considerations

Green synthesis strategies reduce production costs mainly through lowering solvent consumption, template usage, and waste treatment requirements. Solvent-free synthesis minimizes water consumption and simplifies downstream processing while maintaining the crystallinity of zeolite products (Ji F. et al., 2024; Ji and Zhang, 2024; Zhang et al., 2025). Likewise, template-free synthesis eliminates expensive organic structure-directing agents (OSDAs) and avoids the calcination step for template removal, leading to lower material and energy consumption (Bragina et al., 2024; Jia et al., 2021; Li et al., 2021). Seed-assisted template-free synthesis further improves crystallization efficiency and product quality, making this route attractive for future industrial production (Xie et al., 2022; Zhang et al., 2021).

Renewable silicon and aluminum sources, including rice husk ash, fly ash, and industrial solid wastes, provide an economical alternative to commercial silica and alumina while promoting waste recycling (Akinjokun et al., 2024; Flores et al., 2021; Grabias-Blicharz et al., 2022; Liu F. F. et al., 2025). However, pretreatment is generally required to remove impurities and reduce compositional fluctuations. In contrast, ionothermal synthesis offers efficient crystallization under low-pressure conditions because of the negligible vapor pressure of ionic liquids, but the relatively high cost of ionic liquids and the need for solvent recycling remain important economic barriers (Han et al., 2023; Ma et al., 2009; Morris, 2009). Similarly, dry gel conversion (DGC) significantly reduces solvent consumption and waste generation, although precise humidity control and specialized reactors may increase capital investment (Brazovskaya et al., 2025; Naik et al., 2003). Therefore, future studies should combine environmental assessment with techno-economic analysis to identify economically viable green synthesis routes for industrial applications.

Although green synthesis strategies generally reduce environmental impacts by decreasing solvent consumption, template usage, and waste generation, quantitative comparisons remain limited. Existing studies often report synthesis performance under different experimental conditions, making direct comparisons of energy consumption, carbon emissions, and environmental impacts difficult. Future work should establish standardized sustainability metrics, including energy consumption per unit product, solvent utilization efficiency, waste generation, atom economy, carbon footprint, and life-cycle assessment (LCA), to enable objective evaluation of different green synthesis strategies.

### 6.2 Scale-up challenges and opportunities

Although considerable progress has been achieved at the laboratory scale, the industrial implementation of green zeolite synthesis still faces several challenges. For solvent-free and template-free syntheses, homogeneous mixing of solid precursors and uniform heat transfer become increasingly difficult as reactor size increases (Ji F. et al., 2024; Xie et al., 2022). In addition, renewable raw materials such as rice husk ash and fly ash exhibit variable chemical compositions, making pretreatment and quality control necessary to ensure reproducible crystallization (Akinjokun et al., 2024; Grabias-Blicharz et al., 2022; Liu F. F. et al., 2025).

Several recent studies provide encouraging evidence for the scale-up potential of green zeolite synthesis. Seed-assisted template-free synthesis has achieved reduced production costs together with high crystallization efficiency (Li et al., 2021; Zhang et al., 2021), while DGC has demonstrated low-solvent crystallization under controlled humidity and temperature conditions (Brazovskaya et al., 2025; Naik et al., 2003). Progress has also been reported for ionothermal and electrochemical synthesis, although their practical implementation still requires advances in ionic-liquid/electrolyte recycling and reactor design (Morris, 2009; Warty et al., 2024; Yu et al., 2015). In addition, the recent report on the eco-friendly scale-up synthesis of ZSM-5 (Yin et al., 2024) demonstrates that laboratory-scale green synthesis can be transferred toward larger-scale production through appropriate process optimization.

From an industrialization perspective, the technological maturity of these green synthesis routes remains highly uneven. Conventional hydrothermal synthesis is already commercially established, whereas hydrothermal-derived strategies, particularly seed-assisted, template-free, and solvent-reduced routes, may offer relatively straightforward pathways toward scale-up because they can largely utilize existing hydrothermal infrastructure. For alternative synthesis routes, industrial implementation is governed by route-specific engineering constraints: DGC requires effective control of vapor transport and heat/mass transfer, whereas ionothermal and electrochemical synthesis depend strongly on solvent/electrolyte recycling and reactor engineering. These techno-economic characteristics and scale-up considerations are summarized in Table 6. Importantly, standardized quantitative data on productivity, specific energy consumption, template/solvent recovery efficiency, production cost, and carbon footprint remain scarce, making rigorous techno-economic comparison and numerical TRL assignment premature.

**TABLE 6:** Techno-economic advantages, limitations, and scale-up considerations of representative green zeolite synthesis strategies.

Green synthesis strategy	Techno-economic advantages	Economic limitations	Scale-up considerations	Technology maturity	Ref.
Solvent-free synthesis	Eliminates most solvent usage; reduces wastewater treatment and drying costs	High solid mixing requirement; crystallization uniformity must be controlled	Suitable for continuous solid-state processing	Laboratory–pilot/developing	Ji et al. (2024a); Ji and Zhang (2024); Zhang et al. (2025); Zhong et al. (2025)
Template-free synthesis	Eliminates expensive OSDAs and template calcination; lower operating cost	Narrow synthesis window and stricter crystallization control	High potential for industrial production using seed-assisted synthesis	Relatively mature/scale-up demonstrated	Bragina et al. (2024); Jia et al. (2021); Li et al. (2021); Xie et al. (2022); Zhang et al. (2021)
Renewable raw materials	Low-cost feedstocks; waste valorization; reduced consumption of commercial silica/alumina	Pretreatment and compositional variation increase processing cost	Requires feedstock standardization and quality control	Laboratory–pilot/feedstock-dependent	Akinjokun et al. (2024); Flores et al. (2021); Grabias-Blicharz et al. (2022); Liu et al. (2025b); Zhang et al. (2025)
Ionothermal synthesis	Ambient-pressure operation; shortened crystallization (especially with microwave assistance)	Ionic liquids are expensive and require efficient recycling	Industrialization depends on solvent recovery and reuse	Laboratory/emerging	Han et al. (2023); Ma et al. (2009); Morris (2009); Parnham and Morris (2007); Wheatley et al. (2010)
Dry gel conversion	Reduced solvent consumption and waste generation	Additional humidity control and specialized reactors	Suitable for continuous or semi-continuous processing	Laboratory–pilot/developing	Brazovskaya et al. (2025); Naik et al. (2003); Wang and Zhao (2024)
Electrochemical synthesis	Mild reaction conditions; controllable crystal growth	Limited productivity and reactor scale	Requires further pilot-scale validation	Laboratory/emerging	Warty et al. (2024); Yu et al. (2015)

## 7 Machine learning guided zeolite synthesis

The sustainable synthesis strategies discussed in Sections 2–6 have substantially expanded the toolbox for environmentally friendly zeolite production. However, the optimization of these synthesis routes still requires extensive experimental exploration. Recent advances in artificial intelligence (AI) and machine learning (ML) provide new opportunities to establish quantitative relationships between synthesis parameters, framework topology, and material performance, thereby accelerating the rational development of green zeolite synthesis. Zeolite synthesis is a complex process involving multiple parameter adjustments, where the resulting topological structure exhibits strong coupling with synthesis conditions such as raw material composition, temperature, and type of structural directing agent. So far, the International Zeolite Association (IZA) has recorded over 250 different types of frameworks.

**FIGURE 10:**
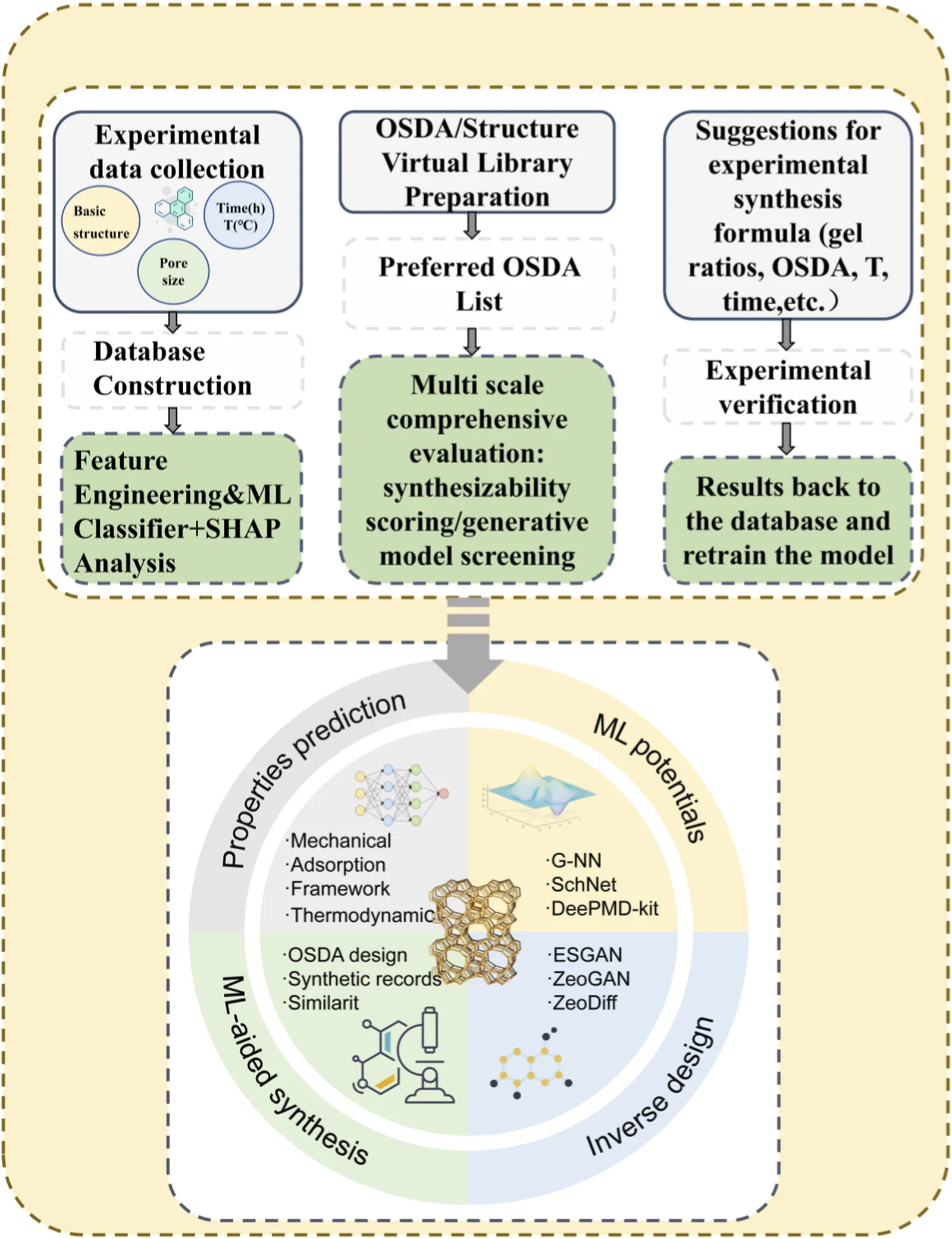
The main process of machine learning for digital design of zeolites.

However, only a small portion of the millions of potential structures predicted theoretically have been successfully synthesized through experiments (Lee et al., 2025). Traditional research on zeolite materials has long relied on experimental trial and error methods, with long development cycles, high costs, and limited understanding of complex crystallization mechanisms (Hasnan et al., 2023; Isahak and Al-Amiery, 2024; Moliner et al., 2019). The development of high-throughput experiments and computational simulations has accumulated a large amount of data for zeolite synthesis, laying the foundation for the application of machine learning. By constructing data-driven models, machine learning can predict the applicability of organic structure directing agents (OSDA), optimize synthesis routes, and screen new structures, significantly improving the efficiency and accuracy of zeolite synthesis (Wu et al., 2025). Machine learning is good at extracting implicit rules from high-dimensional and nonlinear data, especially suitable for dealing with zeolite synthesis, a complex system involving many interrelated variables (such as gel composition, crystallization temperature, time, template structure, etc.) (Hasnan et al., 2020; Moliner et al., 2005). In recent years, researchers have applied ML to multiple stages such as the generation of hypothetical zeolite structures, screening of organic structure directing agents (OSDA), optimization of synthesis parameters, and automatic analysis of X-ray diffraction (XRD) data, forming a preliminary closed-loop research model of “computational prediction experimental verification data feedback”. The main workflow of ML-assisted zeolite design and synthesis is schematically illustrated in Figure 10.

### 7.1 Topology prediction

Accurate prediction of zeolite topology is a prerequisite for target synthesis. By extracting the correlation between structural descriptors and synthesis feasibility, machine learning (ML) can effectively filter out synthesizable frameworks from a vast library of hypothetical structures.

#### 7.1.1 Computational generation of hypothetical zeolite structures

The diversity of zeolite skeleton topology far exceeds the number of synthesized structures, and the systematic generation and screening of hypothetical zeolite structures is the starting point for discovering new materials. Early work was mainly based on enumeration of four connected networks under symmetry constraints, such as the SCIBS method developed by Treacy, which can generate all possible 4-connected graphs under given space groups and independent T atom numbers (Treacy et al., 2004). The Systre program developed by Delgado Friedrichs et al. solved the mathematical problem of isomorphism determination in three-dimensional periodic structure diagrams, laying the foundation for the construction of hypothetical structure databases. With the improvement of computing power, genetic algorithm (GA) has become a powerful tool for generating hypothetical zeolite structures. ZeoGAsolver and other algorithms encode T atomic coordinates into chromosomes, and search for structures that satisfy constraints such as tetrahedral coordination and reasonable bond length and angle in asymmetric units through selection, crossover, mutation, and other operations (Liu et al., 2017). Baumes et al. deployed GA on GPU platforms, significantly improving search efficiency and successfully generating stable structures containing 6-14 independent T atoms. ZEFSAII and FraGen are based on Monte Carlo simulation methods, optimizing atomic positions through simulated annealing or parallel tempering strategies to adapt to the set framework density and diffraction intensity constraints (Falcioni and Deem, 1999).

Research has shown that introducing key structural descriptors, including O-T-O bond angle deviation (ω < 30°), vertex symbols representing 4-, 6-, 8-, and 12 membered ring combinations, and T-O-T bond angles (>130°), can identify 420 potential synthesizable zeolitic imidazolate frameworks (ZIFs) from over 4 million hypothesized structures. Based on the calculation of force field energy, these candidate structures can be further classified into three feasibility levels (Level 1 to Level 3) according to their energy stability. This strategy successfully guided the experimental synthesis of two previously unknown topologies, UZIF-31 and UZIF-33, improving screening efficiency by approximately four orders of magnitude compared to traditional methods and providing an effective paradigm for new topology discoveries (Lee et al., 2025; Razali et al., 2025; Rusdan et al., 2022).

#### 7.1.2 Machine learning prediction of OSDA-Zeolite interactions

Organic structure directing agents (OSDA) determine the formation of specific topological structures during zeolite crystallization through spatial matching and energy stabilization, and their interaction with the zeolite framework has always been an important object of machine learning research. Daeyaert et al. used neural networks with molecular structure descriptors as inputs to predict the stabilization energies of 4781 OSDAs in BEA zeolite, with an accuracy comparable to molecular dynamics. Based on this, they screened a batch of candidate molecules that could be chemically synthesized (Daeyaert et al., 2019). This strategy has been successfully validated experimentally for the directed synthesis of zeolites such as SFW and STW. Hoffmann et al. developed an ensemble descriptor α(i,j,t) using symbolic regression by constructing a quantitative structure-activity relationship (QSAR) model. This descriptor combines the competitive coefficient (C), directional coefficient (D), and binding energy to quantitatively evaluate the compatibility between OSDA and zeolite frameworks. Compared with the traditional template energy descriptor E (i,j,t), the α(i,j,t) descriptor increases the average area under the curve (AUC) for predicting OSDA applicability from 68.3% to 71.8%. More importantly, descriptors effectively capture the synergistic interactions between template molecules and frameworks, as well as the lnD (form,Si) has been identified as the primary factor controlling topological selectivity, providing mechanistic evidence of the crucial role of intermolecular directional interactions in crystal nucleation processes (Hoffman et al., 2025).

Recent studies further indicate that an evaluation framework relying solely on van der Waals interaction energy (EvdW) is insufficient to accurately describe OSDA framework compatibility. On the contrary, the total energy (Etot) and synthesis energy (Esyn) provide a more comprehensive description, which also includes skeletal stability and reaction enthalpy. Based on these concepts, a multi-scale energy fusion machine learning model has been developed to accurately predict the OSDA framework binding energy with an error of less than 5 kJ mol^‒1^, successfully guiding the rational design of efficient structural directing agents for STW and MSE frameworks. In addition, deep learning models trained on 4781 molecular dynamics (MD) datasets were able to quickly screen for stable energies within the β zeolite system. Experimental verification has confirmed that the prediction accuracy is comparable to MD simulation. Among the 469 candidate OSDAs screened, 23 successfully produced highly crystalline β zeolite. Compared with traditional trial and error methods, the screening efficiency has been improved by about two orders of magnitude (Tang et al., 2025).

Although significant progress has been achieved in topology prediction and OSDA-framework matching, the successful synthesis of high-performance zeolites relies not only on accurate computational prediction but also on rational framework engineering and structural optimization. Recent studies have further emphasized that framework engineering and structural optimization provide an important bridge between green synthesis strategies and AI-assisted materials design, enabling the rational optimization of framework topology, pore architecture, and catalytic performance (Noor Zulfiqar et al., 2025).

### 7.2 Synthesis condition optimization

The synthesis conditions of zeolite, including reactant composition, temperature, crystallization time, and alkalinity, have a critical impact on phase purity, topological selectivity, and crystallinity. By establishing a quantitative relationship between synthesis parameters and the final product, machine learning provides an effective framework for intelligent process optimization and high throughput experimental design.

Moliner et al. constructed an artificial neural network (ANN) model using 4 M ratios, Na/(Si + Al), TEA/(Si + Al), OH^−^/(Si + Al), and H_2_O/(Si + Al), as input variables to predict the crystallinity of Beta zeolites and the content of competing phases. The ANN model significantly outperformed conventional quadratic regression models in prediction accuracy (Corma et al., 2005; Mashayekhi et al., 2025). Li et al. compared geometric harmonics, neural networks, and Gaussian process regression for FAU zeolite synthesis and found that the geometric harmonics model exhibited superior performance in predicting products with high Si/Al ratios (Si/Al > 3) (Jensen et al., 2021; Li et al., 2023). Gradient analysis further revealed that reducing the Na_2_O content is the key factor for increasing the framework Si/Al ratio. Based on this insight, an optimized synthesis strategy with prolonged crystallization time successfully produced pure-phase FAU zeolite with a Si/Al ratio of 3.5, representing the highest value achieved through template free direct synthesis.

The establishment of large scale synthesis databases has further promoted the development of synthesis route prediction models. For example, a random forest model trained on the ZeoSyn database, containing 23,961 synthesis routes involving 233 framework types and 921 OSDAs, achieved a prediction accuracy of 73% on the test dataset (Pan et al., 2024). SHapley Additive exPlanations (SHAP) analysis further identified the Na/(Si + Al) molar ratio and crystallization temperature as the most influential thermodynamic variables governing topology selectivity, particularly for MFI and CHA frameworks. These findings provide quantitative guidance for phase selective synthesis and highlight the potential of ML to accelerate synthesis optimization while reducing experimental costs.

### 7.3 Structure characterization

Machine learning has become an important tool for characterizing zeolite structures, enabling automatic analysis of high throughput experimental data and rapid extraction of microstructural information. XRD is the main method for phase identification of zeolites, but manual analysis of high throughput data is extremely time consuming. The clustering algorithm treats each diffraction pattern as a high dimensional vector and automatically classifies it by calculating similarity, which can quickly identify pure, mixed, and amorphous products. PCA can reduce the original diffraction intensity data to 2-3 principal components, and the adaptive time warping (ATW) algorithm solves the problem of peak shift caused by grain size or element substitution, achieving automatic qualitative analysis of multiphase mixtures (Baumes et al., 2009; Corma et al., 2006). Combining infrared spectroscopy, solid state nuclear magnetic resonance and other characterization methods, ML assisted identification of active site types and quantities. In the study of Ru based ammonia synthesis catalysts, MnN (100) was identified as the optimal carrier through a random forest model, and the physical rationality of the model was verified through NH_3_-TPD and H_2_-TPD experiments (Jayarathna et al., 2025).

### 7.4 Challenges and future perspectives

Although machine learning has made significant progress in zeolite synthesis and design, there are still several key challenges that need to be addressed.

#### 7.4.1 Data scarcity and standardization

The performance of ML models is highly dependent on the quantity and quality of training data. However, current zeolite synthesis data is mainly scattered in literature, and there is a lack of unified standards for experimental protocols and metadata records. Moreover, positive results far outnumber negative results, which can easily lead to “survivor bias” in the model. In recent years, natural language processing (NLP) technology and database construction such as Zeobank have promoted automatic data extraction and integration, but experimental data still accounts for less than 30% of all available data. In the future, an open, shared, and standardized high-quality database should be established, and key experimental variables such as precursor purity, aging conditions, and crystallization processes should be systematically recorded to improve the transferability and generalization ability of the model (Jensen et al., 2019; Pan et al., 2024; Wu et al., 2025).

#### 7.4.2 Model interpretability and physical consistency

ML models are often seen as’ black boxes’, and their predicted results lack clear physical and chemical explanations. Although SHAP and permutation importance analysis can reveal some feature contributions, they are still difficult to completely replace kinetic models based on reaction mechanisms. In the future, we should further develop Physical Information Neural Networks (PINNs), Symbolic Regression, and chemically meaningful descriptors (such as α(i,j,t)), combining physical constraints with data driven models to enhance model interpretability and mechanistic understanding while improving prediction accuracy (Hoffman et al., 2025).

#### 7.4.3 Multiscale modeling and multi-objective optimization

Zeolite catalysis involves multi-scale processes from molecular scale (active sites, reaction pathways) to particle scale (mass transfer and diffusion), while existing machine learning models mainly focus on thermodynamic stability, neglecting dynamic factors such as nucleation barriers, crystallization pathways, and intermediate phase evolution. In the future, a cross scale, multi-objective optimization framework that balances thermodynamic stability and kinetic accessibility should be established by combining molecular dynamics simulations, micro dynamics models, computational fluid dynamics (CFD), and machine learning surrogate models, to achieve collaborative design of catalyst composition, structure, and reactor performance (Wijerathne et al., 2024; Wu et al., 2025).

#### 7.4.4 Closed-loop experimental validation

Many current ML studies remain at the computational prediction stage and lack effective feedback from experimental validation. Future developments should focus on self-driving laboratories that integrate ML prediction, robotic synthesis, online characterization, and active learning into a fully automated “design-synthesis-characterization-optimization” workflow. In addition, the emergence of large language models (LLMs), such as GPT, for literature mining and experimental protocol design is expected to further accelerate autonomous materials discovery.

Despite these advances, the application of AI to zeolite synthesis remains subject to several limitations. Current machine-learning models are often trained on relatively small and heterogeneous datasets derived from different experimental conditions, which limits model transferability and predictive reliability. In addition, incomplete reporting of unsuccessful experiments introduces data bias, while differences in precursor composition, synthesis protocols, and characterization methods further complicate data standardization. The limited interpretability of some machine-learning models also makes it difficult to establish clear relationships between synthesis parameters, crystallization mechanisms, and the resulting zeolite structures. Therefore, the development of standardized, high-quality datasets incorporating both successful and unsuccessful synthesis experiments remains essential for reliable AI-assisted zeolite design.

**FIGURE 11:**
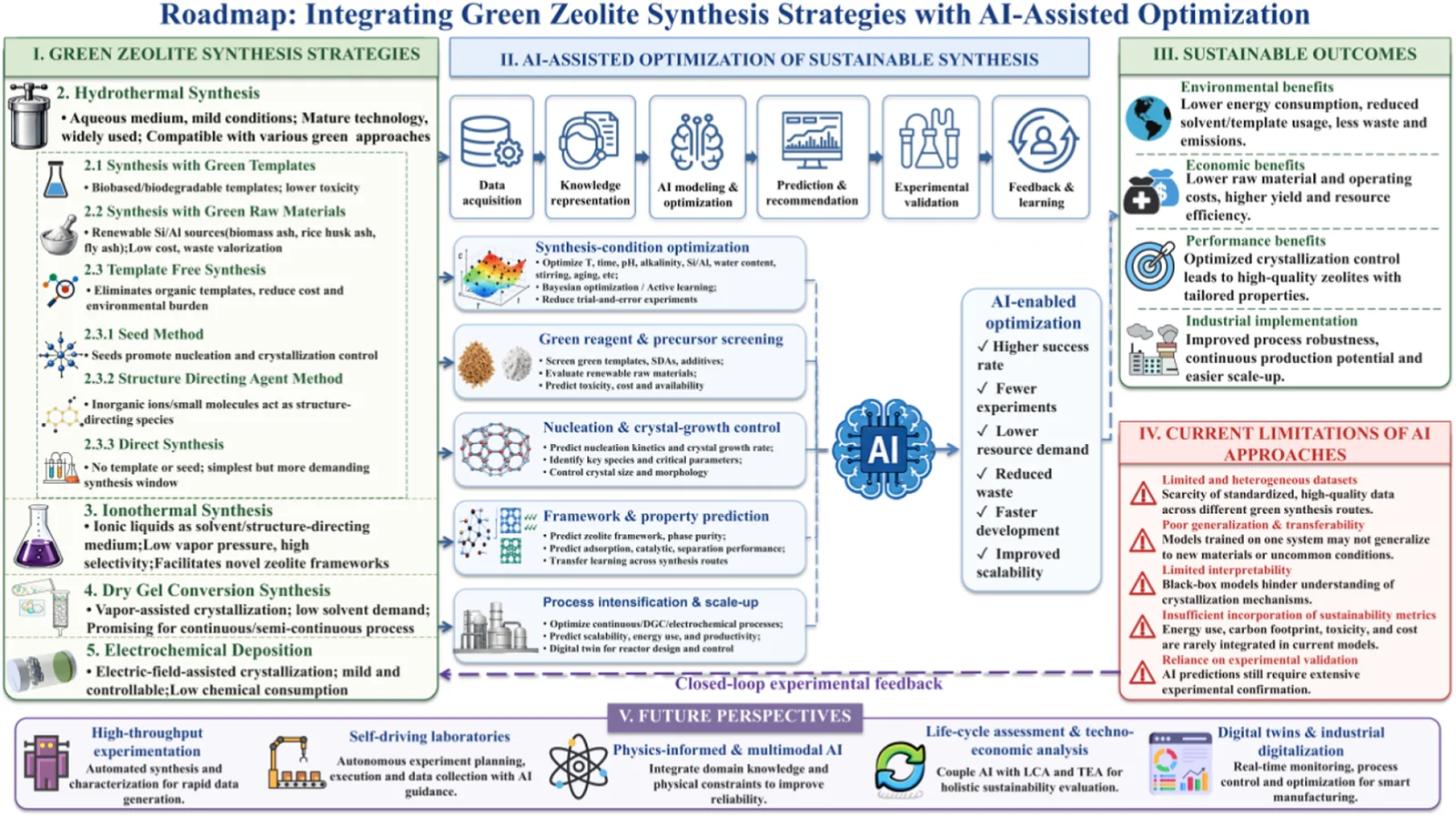
Roadmap: Integrating green zeolite synthesis strategies with AI-assisted optimization.

More importantly, the role of AI in sustainable zeolite synthesis should extend beyond framework topology prediction. Machine-learning models can establish relationships between synthesis conditions and crystallization outcomes, thereby reducing trial-and-error experiments and identifying synthesis windows with lower temperatures, shorter crystallization times, and reduced solvent or template consumption. Data-driven approaches may also facilitate the screening of renewable silica and alumina precursors and the optimization of template-free, solvent-free, ionothermal, DGC, and electrochemical synthesis routes. When coupled with sustainability indicators, techno-economic analysis, and process-scale data, AI could enable multi-objective optimization that simultaneously considers zeolite performance, energy consumption, resource utilization, waste generation, and production cost. Ultimately, integrating machine learning with high-throughput experimentation and closed-loop optimization may provide a pathway from empirical laboratory synthesis toward more resource-efficient, scalable, and sustainable zeolite manufacturing. Based on these considerations, Figure 11 presents an integrated roadmap linking representative green zeolite synthesis strategies with AI-assisted optimization, highlighting their integration from synthesis-condition optimization to process-scale implementation.

## 8 Conclusion

Current zeolite synthesis technologies are relatively mature. However, with growing environmental awareness and increasing emphasis on green chemistry, zeolite synthesis is progressively shifting toward more sustainable routes. Although conventional hydrothermal synthesis offers advantages such as technological maturity and good tunability, it often involves long crystallization times, substantial water consumption and wastewater generation, and relatively high energy demand, depending on the synthesis conditions. Ionothermal synthesis benefits from the low volatility, thermal stability, and tunable physicochemical properties of ionic-liquid media; however, the relatively high cost of ionic liquids and challenges associated with their recovery and purification currently limit large-scale application. Dry gel conversion substantially reduces bulk solvent consumption and wastewater generation, but its crystallization behavior is highly sensitive to humidity, vapor transport, precursor homogeneity, and reactor configuration. Solvent-free synthesis can substantially reduce bulk solvent consumption and simplify the synthesis process; however, challenges related to precursor homogenization, heat and mass transfer, reproducibility, and scale-up remain to be addressed.

Overall, no single synthesis strategy is universally superior. Hydrothermal synthesis remains the most mature route for large-scale production, whereas template-free and solvent-free strategies show considerable potential for reducing resource consumption and waste generation. Ionothermal synthesis enables the preparation of unique framework structures but requires more economical ionic liquid recycling. DGC and electrochemical synthesis are promising emerging technologies, although further studies on process intensification and industrial scale-up are still needed.

Despite these advances, several fundamental and engineering challenges remain unresolved. A deeper understanding of precursor evolution, nucleation, crystal growth, and framework formation under green synthesis conditions is still required to enable rational control of crystallization. Meanwhile, compositional variability of renewable and waste-derived feedstocks, restricted synthesis windows in template-free systems, solvent/template recovery, and maintaining phase selectivity and product quality under low-solvent conditions remain important practical challenges. At larger scales, heat and mass transfer, reactor design, continuous processing, and process reproducibility must also be addressed. Furthermore, standardized quantitative data on energy consumption, resource efficiency, carbon footprint, production cost, and life-cycle impacts are required for rigorous comparison of competing synthesis routes.

AI offers an important opportunity to address these challenges by shifting sustainable zeolite synthesis from empirical trial-and-error toward data-driven optimization. Future efforts should focus on developing standardized and interoperable synthesis databases, using specialized language models to extract synthesis knowledge from the literature, and integrating machine learning with high-throughput and robotic experimentation for closed-loop optimization of synthesis conditions. Physics-informed and multimodal models that incorporate chemical composition, precursor chemistry, crystallization conditions, and structural information will be particularly important for improving model generalizability and interpretability. AI should also be increasingly coupled with techno-economic analysis and life-cycle assessment so that optimization targets extend beyond crystallinity or framework topology to include energy demand, resource consumption, waste generation, carbon footprint, and production cost.

Combining green raw materials with green synthesis methodologies represents another promising direction for the sustainable development of zeolite synthesis. Accordingly, future sustainable zeolite manufacturing should progress along an integrated roadmap from fundamental mechanistic understanding to green synthesis optimization, AI-assisted experimental design, process intensification and scale-up, and finally quantitative techno-economic and life-cycle evaluation. In the longer term, coupling renewable feedstocks and low-impact synthesis routes with self-driving laboratories, digital twins, real-time process monitoring, and closed-loop AI optimization may enable more autonomous and resource-efficient zeolite manufacturing. Achieving this transition will require close integration of green chemistry, crystallization science, data science, reaction engineering, and sustainability assessment rather than optimization of any single synthesis step in isolation.
